# Activated hepatic stellate cell-derived Bmp-1 induces liver fibrosis via mediating hepatocyte epithelial-mesenchymal transition

**DOI:** 10.1038/s41419-024-06437-8

**Published:** 2024-01-12

**Authors:** Sizhe Wan, Xianzhi Liu, Ruonan Sun, Huiling Liu, Jie Jiang, Bin Wu

**Affiliations:** 1 Department of Gastroenterology, The Third Affiliated Hospital of Sun Yat-Sen University, Guangzhou, China; 2 Guangdong Provincial Key Laboratory of Liver Disease Research, Guangzhou, China

**Keywords:** Molecular biology, Liver diseases

## Abstract

Liver fibrosis is a reparative response to injury that arises from various etiologies, characterized by activation of hepatic stellate cells (HSCs). Periostin, a secreted matricellular protein, has been reported to participate in tissue development and regeneration. However, its involvement in liver fibrosis remains unknown. This study investigated the roles and mechanisms of Periostin in phenotypic transition of HSCs and relevant abnormal cellular crosstalk during liver fibrosis. The fate of hepatic stellate cells (HSCs) during liver fibrogenesis was investigated using single-cell and bulk RNA sequencing profiles, which revealed a significant proliferation of activated HSCs (aHSCs) in fibrotic livers of both humans and mice. *αSMA*-TK mice were used to demonstrate that depletion of proliferative aHSCs attenuates liver fibrosis induced by carbon tetrachloride and 3,5-diethoxycarbonyl-1,4-dihydrocollidine. Through integrating data from single-cell and bulk sequencing, *Periostin* was identified as a distinctive hallmark of proliferative aHSC subpopulation. Elevated levels of Periostin were detected in fibrotic livers of both humans and mice, primarily within aHSCs. However, hepatic Periostin levels were decreased along with depletion of proliferative aHSCs. Deficiency of Periostin led to reduced liver fibrosis and suppressed hepatocyte epithelial-mesenchymal transition (EMT). Periostin-overexpressing HSCs, exhibiting a proliferative aHSC phenotype, release bone morphogenetic protein-1 (Bmp-1), which activates EGFR signaling, inducing hepatocyte EMT and contributing to liver fibrosis. In conclusion, Periostin in aHSCs drives their acquisition of a proliferative phenotype and the release of Bmp-1. Proliferative aHSC subpopulation-derived Bmp-1 induces hepatocyte EMT via EGFR signaling, promoting liver fibrogenesis. Bmp-1 and Periostin should be potential therapeutic targets for liver fibrosis.

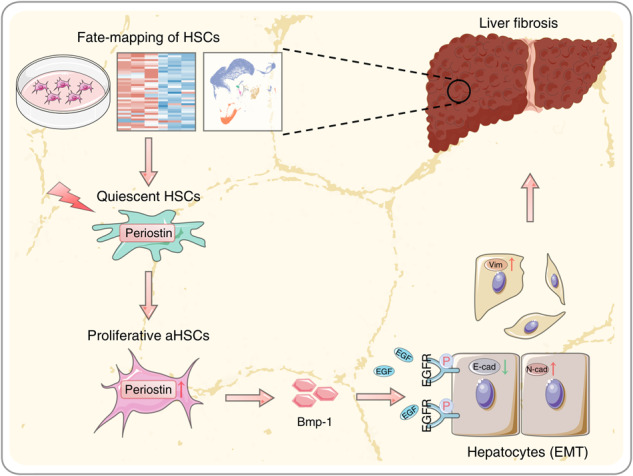

## Introduction

Liver fibrosis is culpable for the evolution of chronic liver diseases to liver cirrhosis and even liver cancer, one of the most common causes of morbidity and mortality worldwide [1, 2]. Upon chronic insult, liver parenchyma is collapse and gradual replacement by excessive amounts of extracellular matrix (ECM), resulting formation of liver fibrosis [3–5]. However, the mechanism underlining fibrogenesis is an ongoing debate. The increasing number of patients with liver fibrosis implies imperative need to develop mechanistic-based therapies.

Hepatic stellate cells (HSCs) are one of the major cell types to contribute liver fibrosis [6, 7]. Following stimulation by fibrotic insult, quiescent HSCs (qHSCs) undergo droplet depletion and transform into activated HSCs (aHSCs), which are capable of producing collagen and ECM [8]. During this process, the proliferation of HSCs significantly contributes to the pool of myofibroblasts [9].

Liver fibrosis involves dynamic cellular communication between parenchymal hepatocytes and non-parenchymal cells, such as HSCs, endothelial cells (ECs), and immune cells. As the primary cell type in the liver, hepatocytes exhibit aberrant crosstalk between ECs and macrophages in response to liver injury [10, 11]. Within the disrupted internal environment, hepatocytes gradually lose their epithelial features and simultaneously acquire mesenchymal features, a process known as epithelial-mesenchymal transition (EMT), which contributes to the liver fibrogenesis [12]. Therefore, elucidating the molecular regulators of hepatocyte EMT and the associated cell crosstalk help uncover the mechanism of liver fibrosis.

Periostin, a secreted matricellular protein, is expressed in collagen-rich fibrous connective tissues and was originally identified as a crucial regulator of bone formation [13, 14]. Recent publications have reported the fibrogenic role of Periostin, demonstrating the significant effect of Periostin-expressing subset of cardiac fibroblasts on myocardial fibrosis [15, 16]. Considering the role of Periostin in the tissue microenvironment due to its secretory properties [17], it is intriguing to explore the cellular communication associated with Periostin in the context of liver fibrosis.

In this study, we unveil a significant increase in proliferative aHSCs characterized by Periostin during liver fibrosis, highlighting their prominent role in the liver fibrogenesis. Mechanistically, bone morphogenetic protein-1 (Bmp-1) is essential involved in the pro-fibrotic communication between Periostin-expressing aHSCs and hepatocytes, consequently promoting the progression of liver fibrosis.

## Materials and methods

### Human sample

Normal liver tissue samples were obtained from the parahemangioma sites of patients with hepatic hemangioma, and paired liver fibrosis samples were collected from patients with hepatitis B virus (HBV)-related liver fibrosis before any therapeutic intervention. Human serum samples were collected from healthy volunteers and patients with liver fibrosis. Informed consent was obtained from all patients prior to their inclusion in the study. The Clinical Research Ethics Committee of the Third Affiliated Hospital of Sun Yat-Sen University approved this procedure. The basic information of the patients examined in this study is summarized in Table S1 and 2.

### Animal studies

*αSMA*-thymidine kinase (*αSMA*-TK) and *Periostin* knockout (*Periostin* KO) mice in the C57BL/6 background were procured from the Jackson Laboratory (Bar Harbor, USA), while C57BL/6 mice were sourced from the Nanjing University Model Animals Institute. The mice were bred to obtain the necessary genotypes for the experiment, and littermates with the wild type phenotype served as the control. All animal protocols were approved by the Institutional Animal Ethics Committee of Sun Yat-Sen University.

### Statistical analysis

Statistical analyses were performed using SPSS (Version 25.0) and R software (Version 4.3.0). A one-way analysis of variance (one-way ANOVA), Student’s *t*-test, the Mann-Whitney rank sum test, and the Mann-Whitney test (unpaired; two-tailed) were used to compare the changes between groups. *p* < 0.05 was considered significant.

The rest of materials and methods are available in the Supplemental Information.

## Results

### Proliferation of activated HSCs occurs in the livers of both humans and mice with liver fibrosis

**Fig. 1:**
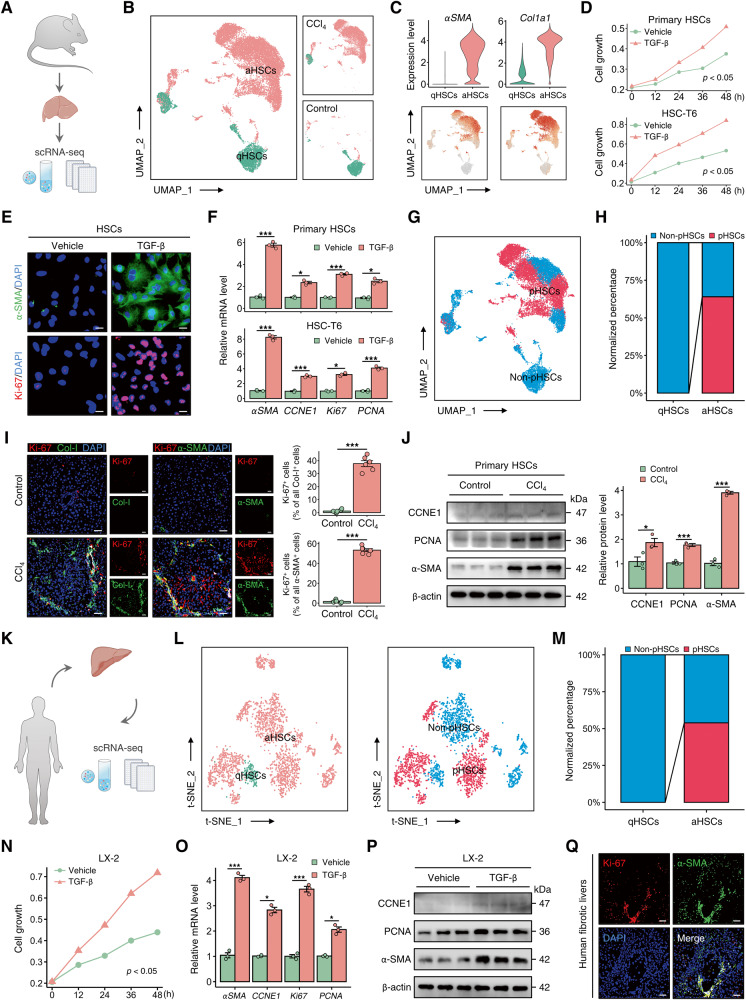
Proliferation of activated HSCs occurs in the livers of both humans and mice with liver fibrosis. **A**, **B** Analysis of mouse liver single-cell RNA sequencing (scRNA-seq) dataset (GSE171904) showed that pool of HSCs obtained from control or CCl_4_-treated mice was classified into two distinct subpopulations: quiescent HSCs (qHSCs) and activated HSCs (aHSCs). **C** Expression and distribution of aHSC markers *αSMA* and *Col1a1* in pooled HSCs obtained from control or CCl_4_-treated mice (GSE171904). **D** CCK-8 assay showed significant proliferation in mouse primary HSCs and HSC-T6 cells after treatment with TGF-β (5 ng/ml). **E** Immunofluorescence staining of α-SMA (green) and Ki-67 (red) in HSC-T6 cells treated with or without TGF-β (5 ng/ml) for 48 h (Scale bar: 25 μm). **F** mRNA levels of *αSMA* and *CCNE1*, *Ki-67*, and *PCNA* (proliferation markers) in mouse primary HSCs and HSC-T6 cells under different treatment conditions. **G** UMAP visualization showed the spatial distribution of proliferative HSCs (pHSCs) within the pool of HSCs obtained from control or CCl_4_-treated mice (GSE171904). **H** Analysis of normalized percentages of non-pHSCs and pHSCs in the qHSCs and aHSCs regions showed that pHSCs constitute more than half of the total aHSC pool in mouse livers (GSE171904). **I** Immunofluorescence staining of Ki-67 (red) and Col-I or α-SMA (green) in liver sections from control or CCl_4_-treated mice. The data were quantified (*n* = 6 per group) (Scale bar: 50 μm). **J** Protein expression levels of CCNE1, PCNA, and α-SMA in primary HSCs isolated from control or CCl_4_-treated mice. The data were quantified (*n* = 3 per group). **K**, **L** Analysis of human liver scRNA-seq dataset (GSE136103) showed the classification of HSC pool obtained from normal controls and liver fibrosis patients into qHSCs and aHSCs, as well as non-pHSCs and pHSCs. **M** Normalized percentages of non-pHSCs and pHSCs were calculated in the qHSCs and aHSCs regions, respectively, of human liver (GSE136103). **N** CCK-8 assay was performed on LX-2 cells treated with or without TGF-β (5 ng/ml). **O**, **P** mRNA and protein levels of α-SMA, CCNE1, Ki-67, and PCNA were downregulated in TGF-β-treated LX-2 cells. **Q** Immunofluorescence staining of Ki-67 (red) and α-SMA (green) in human fibrotic liver samples (Scale bar: 50 μm). All results are shown as mean ± SEM. *p < 0.05; ***p < 0.001. scRNA-seq single-cell RNA sequencing, UMAP uniform manifold approximation and projection, CCl_4_ carbon tetrachloride, qHSCs quiescent HSCs, aHSCs activated HSCs, pHSCs proliferative HSCs, Col-I Collagen-I, CCNE1 Cyclin E1.

To depict the fate-mapping of HSCs, we performed an analysis on a published single-cell RNA sequencing (scRNA-seq) dataset of the mouse liver (GSE171904) [18] (Fig. 1A and Fig. S1A–C). Uniform manifold approximation and projection visualization of HSCs identified two distinct cell subpopulations: quiescent HSCs (qHSCs) and activated HSCs (aHSCs). Notably, the aHSCs demonstrated elevated levels of the activation markers *αSMA* and *Col1a1* [8] (Fig. 1B–C). In accordance with the established dogma [19], aHSCs predominantly localized in the livers of fibrotic mice (Fig. S1D). HSC proliferation is implicated in the expansion of the aHSC population and the development of liver fibrosis [20]. Gene Ontology (GO) enrichment analysis of scRNA-seq data further identified pathways associated with aHSC proliferation (Fig. S1E, F). The CCK-8 assays demonstrated significant proliferation in both primary mouse HSCs and the rat HSC cell line HSC-T6 upon exposure to fibrotic insult (Fig. 1D). After treatment with TGF-β, the expression of the activation marker α-SMA, as well as proliferation markers CCNE1, Ki-67, and PCNA, increased in HSCs, indicating the occurrence of HSC proliferation during their activation (Fig. 1E, F). The analysis of scRNA-seq data revealed that proliferative HSCs (pHSCs), based on known markers of proliferation, were predominantly located in the region of aHSCs instead of qHSCs, constituting more than half of the aHSC population (Fig. 1G–H and Fig. S1G). Immunofluorescence analysis revealed broad co-staining areas of Ki-67, Collagen-I (Col-I), and α-SMA in the fibrotic liver of mice, consistent with the omics results (Fig. 1I). Furthermore, the expression of α-SMA, CCNE1, and PCNA was significantly increased in primary HSCs isolated from CCl_4_-treated mice, confirming the involvement of HSC proliferation in their activation under chronic injury conditions (Fig. 1J). Akin to the results in mouse liver, analysis of the scRNA-seq dataset of normal and fibrotic livers from humans (GSE136103) revealed that pHSCs were primarily localized within the aHSC region (Fig. 1K–M and Fig. S1H–J). In vitro experiments were conducted to confirm the substantial proliferation of LX-2 cells, a human HSC cell line, in response to injury stimuli (Fig. 1N). Treatment with TGF-β significantly upregulated both mRNA and protein levels of α-SMA, CCNE1, and PCNA in LX-2 cells (Fig. 1O, P). Finally, a pronounced colocalization of α-SMA and Ki-67 was detected in fibrotic liver sections obtained from patients (Fig. 1Q). The data indicate a substantial proportion of proliferative aHSCs within the aHSC pool, underscoring their critical role in the expansion of the aHSC population during liver fibrogenesis.

### Depletion of proliferative aHSCs attenuates liver fibrosis

**Fig. 2:**
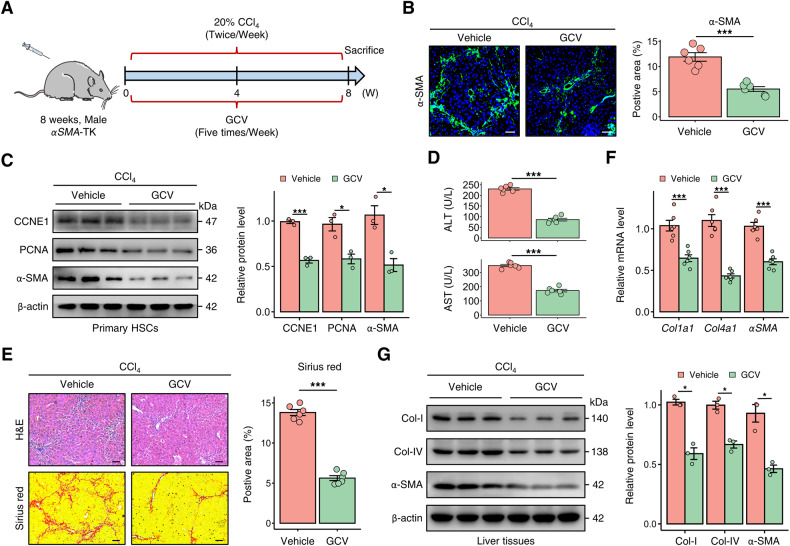
Depletion of proliferative HSCs attenuates liver fibrosis in mice. **A** Ganciclovir (GCV)-administration strategy in *αSMA*-TK mice treated with CCl_4_ to evaluate the impact of proliferative aHSCs on liver fibrogenesis (*n* = 6 per group). **B** Immunofluorescence staining showed a decrease in α-SMA levels in CCl_4_-induced α*SMA*-TK mice treated with GCV. The data were quantified (*n* = 6 per group) (Scale bar: 50 μm). **C** Protein expression levels of CCNE1, PCNA, and α-SMA in primary HSCs isolated from *αSMA*-TK mice treated with or without GCV. The data were quantified (*n* = 3 per group). **D** Serum ALT and AST levels in *αSMA*-TK mice from indicated groups. **E** H&E and Sirius red staining in liver sections of *αSMA*-TK mice from indicated groups. The data were quantified (*n* = 6 per group) (Scale bar: 100 μm). **F**, **G** After treatment with GCV, mRNA and protein levels of Collagen-I (Col-I), Collagen-IV (Col-IV), and α-SMA were reduced in αSMA-TK mice. All results are shown as mean ± SEM. *p < 0.05; ***p < 0.001. TK thymidine kinase, CCl_4_ carbon tetrachloride, GCV ganciclovir, CCNE1 cyclin E1, ALT alanine aminotransferase, AST aspartate aminotransferase, Col-I Collagen-I, Col-IV Collagen-IV.

We utilized *αSMA*-TK transgenic mice to investigate the contribution of proliferative aHSCs to liver fibrosis. These mice allowed for the ganciclovir (GCV)-inducible ablation of proliferating myofibroblasts [21]. In the carbon tetrachloride (CCl_4_)-induced liver fibrosis model, GCV treatment significantly decreased the population of α-SMA-expressing HSCs in the liver of *αSMA*-TK mice (Fig. 2A, B). Primary HSCs isolated from *αSMA*-TK mice treated with GCV showed downregulated expression of α-SMA, CCNE1, and PCNA, confirming the depletion of proliferative HSCs (Fig. 2C). The *αSMA*-TK mice from the GCV group showed improved serum liver function, including alanine aminotransferase (ALT) and aspartate aminotransferase (AST), compared to the control group (Fig. 2D). Moreover, the deficiency of proliferating α-SMA^+^ cells in the liver was associated with a decrease in the fibrotic area, as evidenced by histological staining, and a decline in the expression of fibrotic markers in the liver tissue (Fig. 2E–G). Similarly, in the 3,5-diethoxycarbonyl-1,4-dihydrocollidine (DDC)-administrated *αSMA*-TK mice, the progression of liver fibrosis was hindered due to the decrease in hepatic α-SMA^+^ cells (Fig. S2A–D). These results indicate that deletion of proliferative HSCs hampers liver fibrogenesis induced by various chronic liver injury.

### Periostin is identified as the hallmark of proliferative aHSCs

**Fig. 3:**
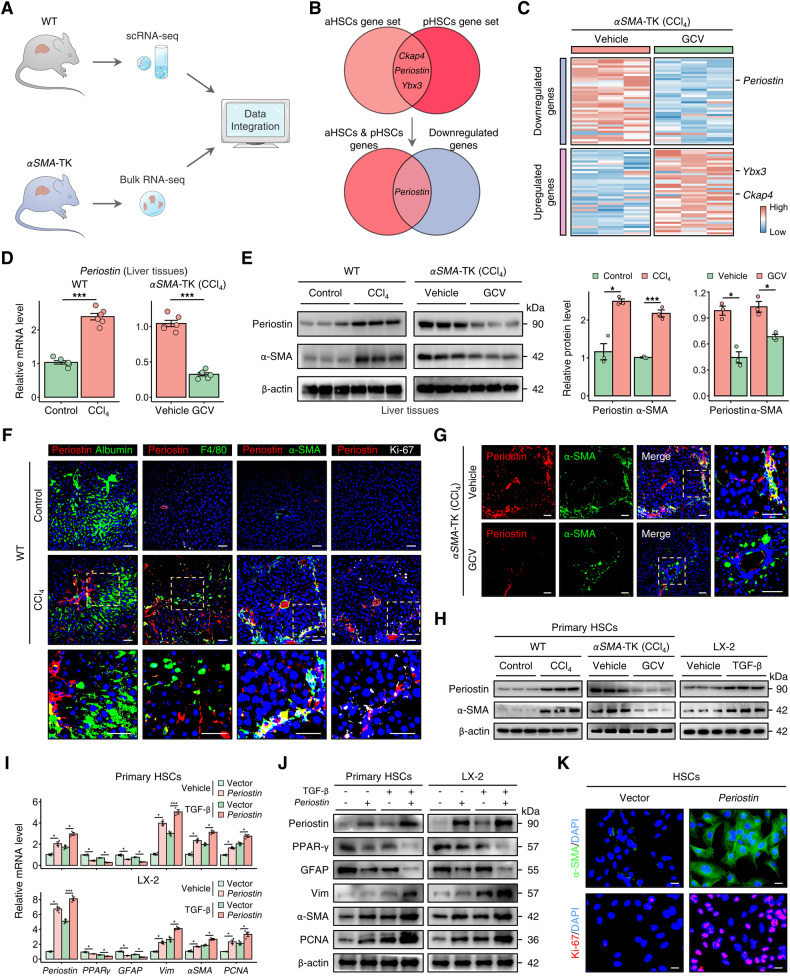
Periostin is identified as the hallmark of proliferative aHSCs. **A** Schematic diagram illustrating the process of marker identification for proliferative aHSCs. This involves integrating liver scRNA-seq data from control or CCl_4_-treated WT mice (GSE171904), as well as liver bulk RNA sequencing data from CCl_4_-induced *αSMA*-TK mice with or without GCV treatment. **B**, **C** Venn plot and heatmap showing that *Ckap4*, *Periostin*, and *Ybx* are genes that overlap in the gene sets of aHSCs and pHSCs. However, only *Periostin* exhibited decreased levels in the bulk liver transcriptomic profile of *αSMA*-TK mice treated with GCV. **D**, **E** mRNA and protein levels of Periostin and α-SMA in liver tissues of WT and *αSMA*-TK mice from indicated groups. **F** Immunofluorescence staining of albumin (hepatocyte marker), F4/80 (monocyte marker), α-SMA (aHSC marker), Ki-67 (proliferation marker), and Periostin in liver sections from WT mice (*n* = 6 per group) (Scale bar: 50 μm). **G** Immunofluorescence staining of Periostin and α-SMA in liver sections from *αSMA*-TK mice (*n* = 6 per group) (Scale bar: 50 μm). **H** Protein expression levels of Periostin and α-SMA were measured in primary HSCs isolated from WT or *αSMA*-TK mice, as well as in LX-2 cells, from indicated groups. The data were quantified (*n* = 3 per group). **I**–**J** mRNA and protein levels of Periostin, PPAR-γ, GFAP, Vimentin (Vim), α-SMA, PCNA in primary HSCs (derived from un-injured mice) and LX-2 cells from the indicated groups. **K** Immunofluorescence staining showed increased expression of α-SMA (green) and Ki-67 (red) in LX-2 cells overexpressing *Periostin* (Scale bar: 25 μm). All results are shown as mean ± SEM. *p < 0.05; ***p < 0.001. WT wild type, TK thymidine kinase; scRNA-seq single-cell RNA sequencing; RNA-seq RNA sequencing; aHSCs activated HSCs; pHSCs proliferative HSCs; CCl_4_ carbon tetrachloride; GCV ganciclovir; Vim, Vimentin.

To gain in-depth insight into the mechanisms by which proliferative aHSCs participate in fibrogenesis, we integrated liver scRNA-seq dataset obtained from control or CCl_4_-treated WT (wild type) mice (GSE171904) and liver bulk RNA sequencing (RNA-seq) profile obtained from CCl_4_-induced *αSMA*-TK mice treated with or without GCV (Fig. 3A). Following a step-by-step filtration of the scRNA-seq data, we selected *Ckap4*, *Periostin*, and *Ybx* as markers that represent HSCs with both proliferative and activated phenotypes for further analysis (Fig. 3B and Fig. S3A, B). However, upon integrating the gene expression profiling data of *αSMA*-TK mice, we observed a downregulation of *Periostin* expression in both the the proliferative aHSC gene set and bulk liver of fibrotic *αSMA*-TK mice treated with GCV (Fig. 3C), indicating *Periostin* as a potential marker for this aHSC subpopulation. The transcriptomic alterations were validated through quantitative real-time polymerase chain reaction (qPCR) and western blotting. The results showed that among these 3 genes, only Periostin was increased in the fibrotic liver of WT mice, while decreased in fibrotic *αSMA*-TK mice treated with GCV (Fig. 3D, E and Fig. S3C, D). At the single-cell level, Periostin was mainly distributed within HSCs, rather than parenchyma and other non-parenchyma cells, according to human and mouse scRNA-seq data (Fig. S3F). Furthermore, compared with controls, the area of Periostin staining was expanded in liver section from CCl_4_-induced mice, and largely co-localised with α-SMA and Ki-67, but not albumin (hepatocyte marker) and F4/80 (monocyte marker), suggesting that Periostin was mainly increased in proliferative and activated HSCs (Fig. 3F). The reduced co-localization of Periostin and α-SMA in the *αSMA*-TK mice confirmed that the Periostin expression was accordingly weakened, as the depletion of proliferative aHSCs (Fig. 3G). In vitro experiments, Periostin expression was higher in LX-2 cells (HSCs) than LO-2 cells (hepatocytes) and THP-1 cells (monocytes), and increased Periostin levels were detected in LX-2 under pro-fibrotic stimuli (Fig. 3H and Fig. S3E). Consistent with the mouse liver tissues, similar change of Periostin expression was observed in primary HSCs isolated from WT and *αSMA*-TK mice (Fig. 3H). Subsequently, we observed activation-like morphology and increased levels of Ki-67, PCNA, Vimentin (Vim), and α-SMA (aHSC markers) in primary HSCs (derived from un-injured WT mice) and LX-2 cells, while PPAR-γ and GFAP (qHSC markers) were decreased after *Periostin* overexpression (Fig. 3I–K and Fig. S3G). This finding demonstrates that Periostin induces the conversion of HSCs from a resting phenotype to a proliferative and activated phenotype, which are distinguishing characteristics of proliferative aHSCs (as depicted in Fig. 1). Overall, these findings suggest that Periostin may serve as a dependable marker for proliferative aHSCs during liver fibrosis.

### Periostin is elevated in liver fibrosis patients

**Fig. 4:**
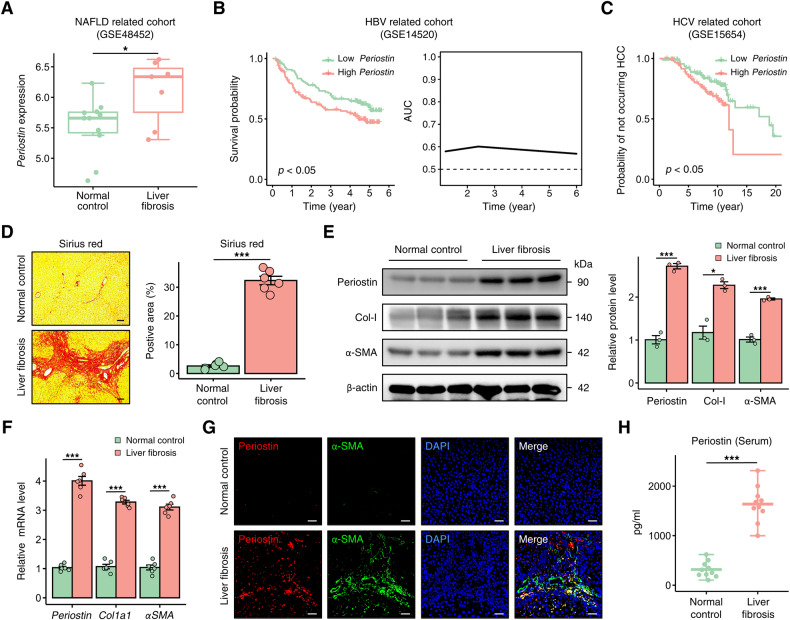
Periostin is elevated in liver fibrosis patients. **A** *Periostin* upregulation in the livers of patients with liver fibrosis in a NAFLD-related cohort. **B** Kaplan–Meier survival curves demonstrated that high *Periostin* levels in the liver were associated with a poorer overall prognosis compared to low Periostin levels in liver fibrosis patients infected with HBV (left). Predictive performance of liver *Periostin* levels in predicting the survival time of these patients with liver fibrosis (right). **C** HCV-infected liver fibrosis patients with high levels of Periostin in the liver exhibited an increased probability of developing HCC. **D** Sirius red staining was used to assess the extent of fibrosis in human samples from indicated groups. The data were quantified (*n* = 6 per group) (Scale bar: 100 μm). **E**, **F** Western blot and qPCR analyses revealed increased levels of Periostin, Col-I, and α-SMA in human liver samples with fibrosis. **G** Immunofluorescence staining showed that elevated Periostin is predominantly localized in α-SMA-positive regions of human fibrotic liver sections (Scale bar: 50 μm). **H** ELISA revealed the serum Periostin levels in normal control individuals and patients with liver fibrosis (*n* = 10 per group). All results are shown as mean ± SEM. *p < 0.05; ***p < 0.001. NAFLD non-alcoholic fatty liver disease, HBV hepatitis B virus; HCV hepatitis C virus; HCC hepatocellular carcinoma; Col-I Collagen-I.

We subsequently conducted validation of Periostin expression in human liver. The analysis of bulk RNA-seq datasets from diverse cohorts showed an elevated expression of *Periostin* in the fibrotic liver of patients with non-alcoholic fatty liver disease (NAFLD) (GSE48452), and higher levels of hepatic *Periostin* were associated with a poor prognosis in liver fibrosis patients infected with HBV (GSE14520) (Fig. 4A, B). In the hepatitis C virus (HCV)-related cohort, elevated levels of *Periostin* in the liver indicate an increased likelihood of adverse outcomes, such as hepatocellular carcinoma (HCC), in patients with liver fibrosis (GSE15654) (Fig. 4C and Fig. S4A). At the single-cell level, *Periostin* expression was predominantly restricted to aHSCs, while minimal expression was observed in qHSCs, as indicated by the human scRNA-seq dataset (GSE136103) (Fig. S4B). Furthermore, human samples were collected to further validate the upregulated mRNA and protein levels of Periostin in fibrotic liver tissues (Fig. 4D–F). The elevated levels of Periostin in fibrotic livers, compared to those in normal controls, were found to be widely distributed in regions positive for α-SMA and Ki-67 (Fig. 4G and Fig. S4C). Importantly, elevated levels of Periostin were detected in the serum of patients with liver fibrosis (Fig. 4H), indicating a strong correlation between Periostin and the progression of liver fibrosis.

### Periostin deficiency attenuates liver fibrosis by inhibiting the proliferation of HSCs

**Fig. 5:**
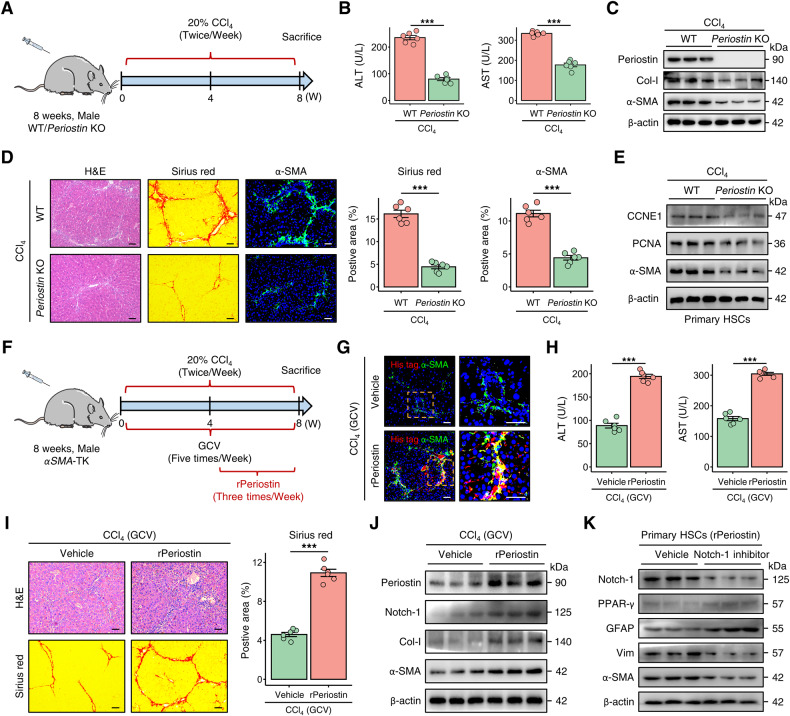
Periostin is implicated in the development of liver fibrosis in mice. **A** Schematic overview illustrating the construction process of the CCl_4_-induced liver fibrosis model in WT and *Periostin* knockout (*Periostin* KO) mice (*n* = 6 per group). **B**, **C** Serum levels of ALT and AST, as well as the protein levels of Periostin, Col-I, and α-SMA in liver tissues, were measured in the indicated groups. **D** H&E, Sirius red, and α-SMA staining demonstrated that the absence of Periostin mitigated liver fibrosis in murine models induced by CCl_4_. The data were quantified (*n* = 6 per group) (Scale bar: 50 μm). **E** Western blot analyses indicated decreased levels of CCNE1, PCNA, and α-SMA in primary HSCs isolated from *Periostin* KO mice treated with CCl_4_. **F** Schematic overview illustrating the experimental strategy of administering recombinant Periostin-His tagged protein (rPeriostin) in CCl_4_-induced *αSMA*-TK mice treated with GCV (*n* = 6 per group). **G** Immunofluorescence staining demonstrated the rPeriostin (red) were internalized by the elevated population of aHSCs (green) in the liver of α*SMA*-TK mice treated with rPeriostin (Scale bar: 50 μm). **H** Serum ALT and AST levels were measured in indicated groups. **I** H&E and Sirius red staining in liver sections of *αSMA*-TK mice from indicated groups. The data were quantified (*n* = 6 per group) (Scale bar: 50 μm). **J** Protein expression levels of Periostin, Notch-1, Col-I, and α-SMA in liver tissues from *αSMA*-TK mice from the indicated groups. **K** Protein expression levels of Notch-1, PPAR-γ, GFPA, Vim, and α-SMA in rPeriostin treated-primary HSCs (isolated from un-injured WT mice) with or without administration of Notch-1 inhibitor (10 μM). All results are shown as mean ± SEM. *p < 0.05; ***p < 0.001. WT wild type, KO knockout, CCl_4_ carbon tetrachloride, ALT alanine aminotransferase, AST aspartate aminotransferase, Col-I Collagen-I, CCNE1 cyclin E1, TK thymidine kinase, GCV ganciclovir, Vim Vimentin.

We then investigated the role of Periostin in liver fibrosis using WT and *Periostin* KO mice. After chronic CCl_4_ injury, the serum levels of ALT and AST were found to be improved in *Periostin* KO mice (Fig. 5A, B). Compared to WT mice, *Periostin* KO mice exhibited reduced collagen deposition and downregulated expression of Col-I and α-SMA in liver tissue (Fig. 5C, D). Furthermore, absence of Periostin impeded the proliferation of HSCs in liver fibrotic mice (Fig. 5E). Similarly, in the DDC-induced fibrosis model, *Periostin* KO mice exhibited reduced liver fibrosis (Fig. S5A–C). Interestingly, the attenuated fibrosis in Periostin-deficient mice was reversed after obtaining exogenous Periostin (Fig. S6). These results provide evidence that the deficiency of Periostin mitigates liver fibrosis through the inhibition of HSC proliferation and activation.

### Periostin reverses attenuation of liver fibrosis in mice with deficient proliferative aHSCs

A recombinant Periostin-His tagged protein (rPeriostin) was administered to CCl_4_-induced *αSMA*-TK mice via tail vein injection, concomitant with GCV treatment (Fig. 5F). Immunofluorescence staining showed that treatment with rPeriostin led to an increase in an augmentation of aHSCs (α-SMA) in the livers of *αSMA*-TK mice. Concurrently, a substantial co-localization of rPeriostin His-tag and α-SMA was observed, indicating the internalization of Periostin by aHSCs (Fig. 5G). *αSMA*-TK mice in the rPeriostin group exhibited elevated serum levels of ALT and AST (Fig. 5H). We observed that administration of rPeriostin abrogated the improvement of liver fibrosis in GCV-treated *αSMA*-TK mice (Fig. 5I, J and Fig. S7A). Nothc-1 is a potential downstream receptor for Periostin [22]. Treatment with rPeriostin led to an elevation in Notch-1 levels, accompanied by substantial co-localization with the rPeriostin in the liver tissue of *αSMA*-TK mice (Fig. 5J and Fig. S7A, B). However, suppression of Notch-1 abolished the conversion of qHSCs to aHSCs induced by Periostin (Fig. 5K and Fig. S7C). Additionally, consistent results were detected in the DDC mouse models (Fig. S5D–F), highlighting a significant association between Periostin and proliferative aHSCs in liver fibrogenesis.

### Periostin-expressing HSC-derived Bmp-1 induces epithelial-mesenchymal transition in hepatocytes

**Fig. 6:**
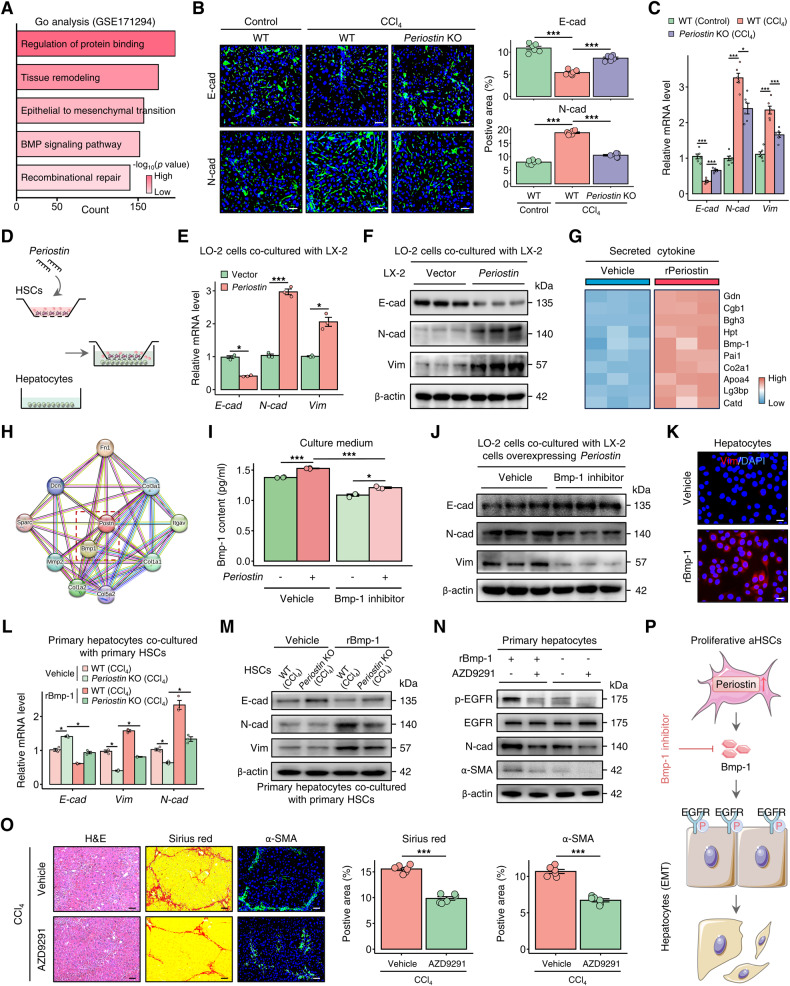
Periostin-expressing HSC-derived Bmp-1 induces epithelial-mesenchymal transition in hepatocytes. **A** GO enrichment analysis of RNA-sequencing dataset from normal control and human fibrotic liver tissues. **B**, **C** Immunofluorescence staining and qPCR were performed to examine the expression of epithelial marker E-cadherin (E-cad), and mesenchymal markers N-cadherin (N-cad) and Vimentin (Vim) in livers of WT and *Periostin* KO mice treated with or without CCl_4_.The data were quantified (*n* = 6 per group) (Scale bar: 50 μm). **D** Schematic diagram illustrating the co-culture of hepatocytes with HSCs transfected with vector or *Periostin*-overexpressing lentivirus. **E**, **F** The levels of E-cad were downregulated, while N-cad and Vim were upregulated in LO-2 cells co-cultured with *Periostin*-overexpressing LX-2 cells at 72 h. **G** Secreted protein profile analysis of culture medium from LX-2 cells treated with or without rPeriostin. **H** Protein-protein interaction network of Periostin was constructed by utilizing STRING database. **I** ELISA showed the Bmp-1 content in the culture medium of LX-2 transfected with vector or *Periostin*-overexpressing lentivirus, and meanwhile these cells were treated with or without Bmp-1 inhibitor (10 μM). **J** Protein expression levels of E-cad, N-cad, and Vim in LO-2 cells co-cultured with *Periostin*-overexpressing LX-2 in indicated groups. **K** Immunofluorescence staining of Vim in LO-2 treated with or without recombinant Bmp-1-His tagged protein (rBmp-1) (10 μM) (Scale bar: 25 μm). **L**, **M** Primary hepatocytes (isolated from un-injured WT mice) co-cultured with primary HSCs isolated from CCl_4_-induced *Periostin* KO mice exhibited upregulated levels of E-cad and downregulated levels of N-cad and Vim when compared to CCl_4_-WT mice, and these effects were blunted by treatment with rBmp-1 (*n* = 3 independent experiments). **N** Western blot analysis for EGFR, p-EGFR, N-cad, and α-SMA in primary hepatocytes (isolated from un-injured WT mice) with or without treatment of rBmp-1 and EGFR inhibitor (AZD9291, 10 μM) (*n* = 3 independent experiments). **O** H&E and Sirius red staining demonstrated that treatment with AZD9291 attenuated liver fibrosis in mice after chronic CCl_4_ injury. The data were quantified (*n* = 6 per group) (Scale bar: 50 μm). **P** Periostin-expressing proliferative aHSCs release Bmp-1, which activates the EGFR signalling, inducing hepatocyte EMT and contributing to liver fibrogenesis. All results are shown as mean ± SEM. *p < 0.05; ***p < 0.001. GO Gene Ontology, CCl_4_ carbon tetrachloride, KO knockout, E-cad E-cadherin, N-cad N-cadherin, Vim Vimentin, rBmp-1 recombinant Bmp-1-His tagged protein, EMT epithelial-mesenchymal transition, aHSCs activated HSCs.

The hepatocyte EMT plays a crucial role in the progression of liver fibrosis [23, 24], as demonstrated by GO enrichment analysis of RNA sequencing data obtained from a publicly available dataset of human fibrotic liver tissue (GSE171294) (Fig. 6A). Consistent with the functional enrichment result, we observed that EMT, characterized by decreased expression of the epithelial marker E-cadherin (E-cad) and increased expression of the mesenchymal markers N-cadherin (N-cad) and Vim, occurred in the livers of CCl_4_-induced mice. However, in the absence of Periostin, the reversal of this EMT and suggests that Periostin plays a significant role in this process (Fig. 6B, C and Fig. S8A). The dysregulation of cellular communication between HSCs and hepatocytes during liver fibrosis has been previously confirmed in our research [25]. In addition, Cellchat analysis from scRNA-seq data (GSE171904) uncovered potential crosstalk between proliferative HSCs and hepatocytes in the fibrotic liver (Fig. S8B). Therefore, we performed a co-culture experiment where we observed the downregulation of E-cad and the upregulation of N-cad and Vim in hepatocytes co-cultured with *Periostin*-overexpressing HSCs (representing the phenotypes of proliferative aHSCs) as the culture time increased (Fig. 6D–F and Fig. S8C). Proteomics of culture medium from rPeriostin-treated HSCs and protein-protein interaction analysis indicated a significant association between Periostin and Bmp-1, a member of the Bmp family (Fig. 6G–H and Fig. S8D, E). Bmp family plays a crucial role in cell crosstalk, causing the phenotypic transition of hepatocytes [12, 26]. Blocking Bmp-1, which was found to be elevated in HSCs following an increase in Periostin, resulted in co-cultured hepatocytes surprisingly regaining their epithelial properties (Fig. 6I–J and Fig. S8F–I). Treatment with recombinant Bmp-1-His tagged protein (rBmp-1) induced the expression of Vim and α-SMA in hepatocytes, which subsequently enhanced the activation of HSCs (Fig. 6K and N and Fig. S8J, K). Furthermore, primary hepatocytes (isolated from un-injured control mice) co-cultured with primary HSCs isolated from CCl_4_-induced mice exhibited EMT, while co-culturing them with primary HSCs derived from fibrotic *Periostin* KO mice suppressed hepatocyte EMT. The addition of exogenous Bmp-1 reversed this suppression (Fig. 6L–M and Fig. S8L). We further revealed that the EGFR, which serves as a critical epidermal growth factor receptor in the EMT of hepatocytes [27, 28], demonstrated increased expression in hepatocytes treated with rBmp-1 (Fig. S8M). The mesenchymal properties of hepatocytes induced by Bmp-1 were abolished by AZD9291, an EGFR inhibitor (Fig. 6N). Interesting, AZD9291 treatment attenuated liver fibrosis in mice induced by chronic CCl_4_ injury (Fig. 6O). Our findings suggest that Periostin-expressing proliferative aHSCs release Bmp-1, which activates the EGFR signalling, inducing hepatocyte EMT and contributing to liver fibrogenesis (Fig. 6P).

### Bmp-1 exacerbates liver fibrosis in *Periostin*-deficient mice through hepatocyte epithelial-mesenchymal transition

**Fig. 7:**
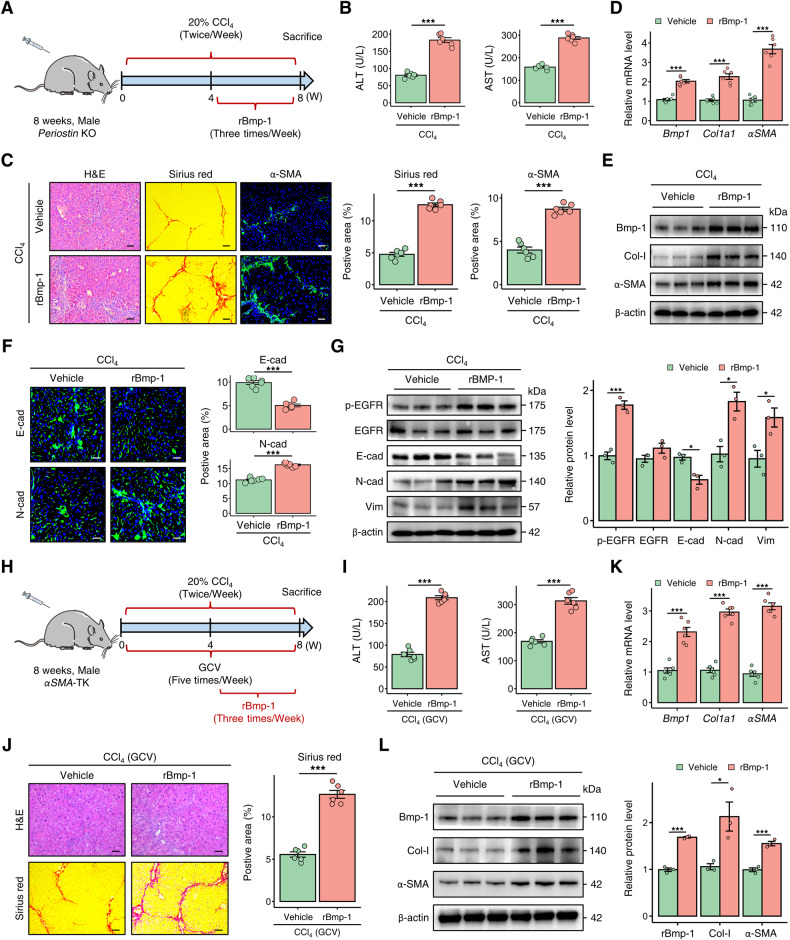
Bmp-1 plays a crucial role in the progression of liver fibrosis in mice. **A** Schematic overview depicting the administration strategy of rBmp-1 in *Periostin* KO mice treated with CCl_4_ (*n* = 6 per group). **B** Serum ALT and AST levels in *Periostin* KO mice treated with or without rBmp-1. **C** H&E, Sirius red, and α-SMA staining demonstrated the reversal of liver fibrosis attenuation in *Periostin* KO mice following treatment with rBmp-1. The data were quantified (*n* = 6 per group) (Scale bar: 50 μm). **D**, **E** qPCR and western blot analysis demonstrated that treatment with rBmp-1 significantly upregulated the levels of Bmp-1, Col-I, and α-SMA in liver tissues of CCl_4_-induced *Periostin* KO mice. **F** Immunofluorescence staining of E-cad and N-cad in liver sections of *Periostin* KO mice from different groups. The data were quantified (*n* = 6 per group) (Scale bar: 50 μm). **G** Protein expression levels of EGFR, p-EGFR, E-cad, and Vim in liver tissues of *Periostin* KO mice from different groups. The data were quantified (*n* = 3 per group). **H** Schematic overview illustrating the experimental strategy of administering rBmp-1 in CCl_4_-induced *αSMA*-TK mice treated with GCV (*n* = 6 per group). **I** Serum ALT and AST levels in *αSMA*-TK mice treated with or without rBmp-1. **J** H&E and Sirius red staining in liver sections of *αSMA*-TK mice from indicated groups. The data were quantified (*n* = 6 per group) (Scale bar: 50 μm). **K**, **L** The levels of Bmp-1, Col-I, and α-SMA were upregulated in liver tissues of *αSMA*-TK mice, following rBmp-1 treatment. All results are shown as mean ± SEM. *p < 0.05; ***p < 0.001. KO knockout, CCl_4_ carbon tetrachloride, rBmp-1 recombinant Bmp-1-His tagged protein, ALT alanine aminotransferase, AST aspartate aminotransferase, Col-I Collagen-I, E-cad E-cadherin, N-cad N-cadherin, Vim Vimentin, TK thymidine kinase, GCV ganciclovir.

To investigate the role of Bmp-1 in Periostin-regulated liver fibrogenesis, we administered rBmp-1 to *Periostin* KO mice via tail vein injection, along with intraperitoneal injection of CCl_4_ (Fig. 7A). The administration of rBmp-1 led to an increase in serum ALT and AST levels in fibrotic *Periostin* KO mice (Fig. 7B). In rBmp-1-treated *Periostin* KO mice with fibrosis, there was an increase in histological injury, inflammatory infiltration, collagen deposition, and expression of fibrotic markers (Fig. 7C–E). In line with the exacerbated liver fibrosis and elevated Bmp-1 levels, the expression of hepatocyte EMT markers, including N-cad, Vim, and EGFR was increased, while E-cad expression decreased in the livers of *Periostin*-deficient mice (Fig. 7F–G). Furthermore, liver fibrosis was alleviated in DDC-induced *Periostin* KO mice after administering rBmp-1 (Fig. S9A–D). The findings provide evidence of the essential role of Bmp-1 in Periostin-provoked liver fibrosis through hepatocyte EMT.

### Bmp-1 aggravates liver fibrosis in *αSMA*-TK mice

To further investigate the involvement of Bmp-1 in liver fibrosis mediated by Periostin-expressing aHSC subpopulation, rBmp-1 was administered to *αSMA*-TK mice induced with CCl4 through tail vein injection, concomitant with GCV treatment (Fig. 7H). Following rBmp-1treatment, the serum liver function indicators, collagen deposition, and fibrotic marker levels were deteriorated in *αSMA*-TK mice. Intriguingly, treatment with rBmp-1 reverses the mitigation of serum liver function and liver fibrosis in *αSMA*-TK mice (Fig. 7I–L). Similarly, an exacerbation of liver fibrosis was observed in DDC-induced *αSMA*-TK mice treated with rBmp-1 (Fig. S9E–H).

### Dabrafenib alleviates liver fibrosis in murine models by targeting Periostin

**Fig. 8:**
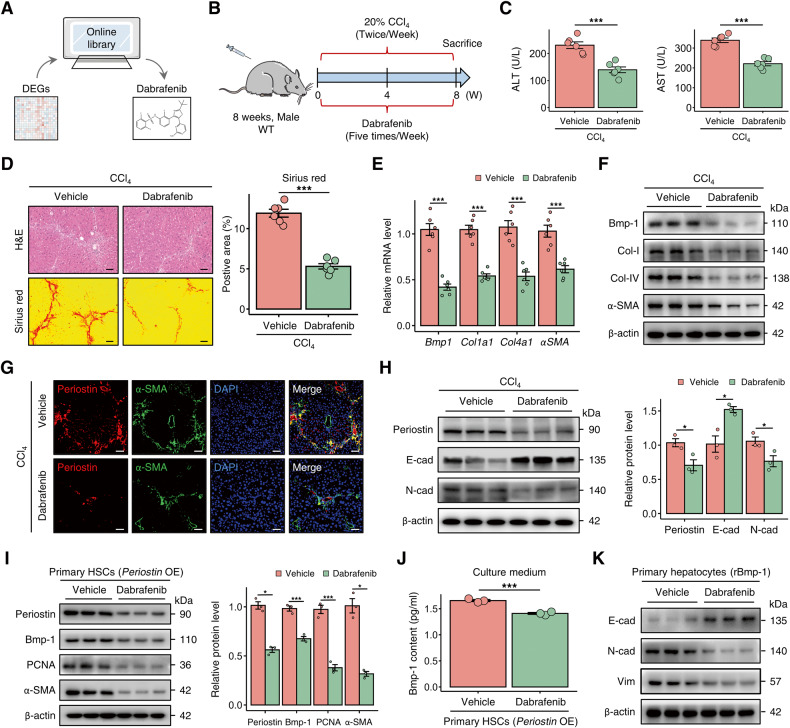
Dabrafenib alleviates liver fibrosis by targeting Periostin. **A** Dabrafenib was identified as potential target compound using the differentially expressed genes in HSCs following *Periostin* perturbation. **B** Schematic overview of the experimental setup for assessing the efficacy of dabrafenib in a CCl_4_-induced liver fibrosis mouse model (*n* = 6 per group). **C** Dabrafenib treatment significantly improved the serum levels of ALT and AST in mice with CCl_4_-induced liver fibrosis. **D** Dabrafenib treatment in CCl_4_-induced mice resulted in reduced liver fibrosis, as indicated by H&E and Sirius red staining. The data were quantified (*n* = 6 per group) (Scale bar: 100 μm). **E**, **F** mRNA and protein levels of Bmp-1, Col-I, Col-IV, and α-SMA in liver tissues of mice from different groups. **G** Immunofluorescence staining of Periostin and α-SMA in liver sections of mice from different groups (Scale bar: 50 μm). **H** Protein expression levels of Periostin, Bmp-1, and N-cad decreased, while levels of E-cad increased in the liver tissues of CCl_4_-induced mice treated with dabrafenib. The data were quantified (*n* = 3 per group). **I** Protein expression levels of Periostin, Bmp-1, PCNA, and α-SMA in *Periostin*-overexpressing primary HSCs (isolated from un-injured mice) treated with or without dabrafenib. **J** ELISA showed the Bmp-1 content in the culture medium of LX-2 cells under different treatment conditions. **K** Protein expression levels of E-cad, N-cad, and Vim in rBmp-1-treated primary hepatocytes (isolated from un-injured mice) in the indicated groups. All results are shown as mean ± SEM. *p < 0.05; ***p < 0.001. DEGs differentially expressed genes, WT wild type, CCl_4_ carbon tetrachloride, ALT alanine aminotransferase, AST aspartate aminotransferase, Col-I Collagen-I, Col-IV Collagen-IV, E-cad E-cadherin, N-cad N-cadherin, OE overexpression, rBmp-1 recombinant Bmp-1-His tagged protein.

We subsequently sought to identify Periostin-targeting antifibrotic treatments that could perturb pro-fibrotic HSC subset. After utilizing the differentially expressed genes of HSCs, derived from *Periostin* perturbation, as a query, we computationally scrutinized the chemogenomic data of transcriptomics in the Cmap database. As a result, dabrafenib was identified as the most suitable candidate compound (Fig. 8A). To more precisely delineate its therapeutic effects, we administered dabrafenib treatment to mice with liver fibrosis (Fig. 8B). Surprisingly, dabrafenib significantly improved liver function and reduced liver fibrosis in mice (Fig. 8C–F). In comparison to those in mice in the control group, fibrotic mice treated with dabrafenib exhibited decreased Periostin, Bmp-1, and α-SMA in the liver, along with attenuated EMT (Fig. 8E–H). Similar results were observed in vitro (Fig. 8I–K and Fig. S10). The livers of DDC-induced mice treated with dabrafenib also showed a comparable reduction in fibrosis (Fig. S11). The data suggest that dabrafenib has the potential to reverse liver fibrosis by inhibiting Periostin.

## Discussion

This study investigated the role and underlying mechanism of Periostin-expressing proliferative aHSCs the progression of liver fibrosis. Through the analysis of scRNA-seq transcriptomics and utilization of transgenic mice models, we have obtained insights that shed light on the pivotal role of proliferative aHSCs in liver fibrogenesis. Periostin was subsequently identified as the hallmark of proliferative aHSCs. It plays a crucial role in mediating the pro-fibrotic communication between HSCs and hepatocytes through the involvement of Bmp-1, thereby influencing the development of liver fibrosis.

HSCs undergo a distinct phenotypic shift during the process of liver fibrogenesis [20]. Through analysis of scRNA-seq data obtained from human and mouse livers, we discovered that approximately half of the aHSCs displayed a proliferative phenotype during liver fibrosis, while almost all pHSCs exhibited activation. This finding indicates that pHSCs are a pivotal subset within the population of aHSCs, supported by results obtained from fibrotic livers of both humans and mice. Furthermore, to further elucidate the role of the aHSC subset, we utilized *αSMA*-TK transgenic mice and demonstrated their crucial involvement in driving the progression of liver fibrosis.

Due to the current lack of a specific marker for proliferative aHSCs, we identified Periostin as the hallmark of this particular subpopulation through the integration of multi-omics data. Abnormal expression of Periostin has been reported in various fibrotic tissues, including the heart, skin, and kidney, attributed to its involvement in tissue injury repair [16, 29–32]. However, studies focusing on the role of Periostin in liver fibrosis remain limited. In line with this finding, we also observed elevated expression of Periostin in the fibrotic livers of both humans and mice, predominantly localized in aHSCs expressing α-SMA, particularly those in a proliferative state. The overexpression of Periostin in HSCs led to the acquisition of a proliferative and activated phenotype that closely resembled the characteristics of proliferative aHSCs. Interestingly, upon the deletion of proliferative HSCs, the expression of Periostin was found to be reduced in the livers of *αSMA*-TK liver fibrosis mice, further supporting the significant association between Periostin and proliferative aHSCs. By utilizing *Periostin* KO mice, we demonstrated that the absence of Periostin markedly reduced liver fibrosis. Moreover, dabrafenib exhibited antifibrotic effects by targeting Periostin.

A meaningful discovery arisen from our study is that Bmp-1 serves as a pivotal mediator of pro-fibrotic communication between Periostin-expressing aHSCs and hepatocytes. Liver cells, including parenchymal hepatocytes, non-parenchymal HSCs, and ECs, demonstrate aberrant cellular communication within the disrupted microenvironment of liver fibrosis [10, 33]. In previous research, we uncovered dysregulation in the crosstalk between HSCs and hepatocytes in the context of liver fibrosis [25]. Here, we have further confirmed the crosstalk between proliferative subpopulation of aHSCs and hepatocytes.Notably, there was a significant correlation between hepatocyte EMT and the expression of Periostin in fibrotic livers. The co-culture experiments revealed that Periostin-overexpressing HSCs were capable of inducing EMT in hepatocytes. Bmp-1 assumes a crucial function in this process by activating EGFR signalling in hepatocytes.Additionally, in fibrotic mice with a deficiency in Periostin, the reduction in liver fibrosis was counteracted by the introduction of exogenous Bmp-1, which coincided with the progression of EMT. These results demonstrate that Periostin-expressing aHSCs release Bmp-1 to induce EMT in hepatocytes, consequently facilitating the progression of liver fibrosis. The specific role and potential mechanism of hepatocytes in liver fibrogenesis after acquiring mesenchymal features, especially their ability to enhance the activation of HSCs, need to be explored. Further investigation in future studies is necessary to elucidate the mechanism of Bmp-1 release by Periostin-expressing aHSCs.

In conclusion, our study provides the proof of concept that proliferative aHSCs, characterized by Periostin, significantly contribute to liver fibrogenesis. Periostin in aHSCs drives their acquisition of a proliferative phenotype and the release of Bmp-1. Proliferative aHSC-derived Bmp-1 inducing hepatocyte EMT through EGFR signalling, thereby promoting liver fibrogenesis. These findings provide insights into a unique perspective on the potential mechanism of liver fibrosis, and targeting of Bmp-1 and Periostin to normalize pro-fibrotic cellular crosstalk presents a promising therapeutic strategy for patients with liver fibrosis.

### Supplementary information

Supplemental Material

Original western blots

Aj-checjlist

## Data Availability

All data generated or analyzed during this study are included in this published article and its supplementary information files. The raw sequencing data will be deposited in Gene Expression Omnibus.
