# Supplementary material for: Activated hepatic stellate cell-derived Bmp-1 induces liver fibrosis via mediating hepatocyte epithelial-mesenchymal transition
Source: Cell Death Dis. 2024 Jan 12;15(1):41. doi: 10.1038/s41419-024-06437-8 (PMC10786946; doi:10.1038/s41419-024-06437-8)
Supplement: Supplementary file 1 — Supplemental Material [file 41419_2024_6437_MOESM1_ESM.docx]

**Supplementary Information**

**Activated hepatic stellate cell-derived Bmp-1 induces liver fibrosis via mediating hepatocyte epithelial-mesenchymal transition**

Sizhe Wan^1,2†^, Xianzhi Liu^1,2†^, Ruonan Sun^1,2^, Huiling Liu^1,2^, Jie Jiang^1,2^, Bin Wu^1,2*^

^1^Department of Gastroenterology, The Third Affiliated Hospital of Sun Yat-Sen University, Guangzhou, China

^2^Guangdong Provincial Key Laboratory of Liver Disease Research, Guangzhou, China

^†^ SW and XL contributed equally to this study.

**Table of contents**

[Supplementary materials and methods 3](#_Toc150547525)

[Animal studies 3](#_Toc150547526)

[Histological staining 4](#_Toc150547527)

[Measurement of serum liver function indices. 4](#_Toc150547528)

[Isolation of primary hepatocytes and HSCs 4](#_Toc150547529)

[Cell culture and transfection 5](#_Toc150547530)

[Co-culture of HSCs with hepatocytes 6](#_Toc150547531)

[RNA extraction and quantitative real-time PCR 6](#_Toc150547532)

[RNA sequencing 6](#_Toc150547533)

[Tandem Mass Tag (TMT) quantitative nanoLC-MS/MS proteomics 7](#_Toc150547534)

[Western blotting analysis 8](#_Toc150547535)

[Construction of recombinant protein 8](#_Toc150547536)

[Enzyme-linked immunosorbent assay (ELISA) 9](#_Toc150547537)

[Data collection and analysis 9](#_Toc150547538)

[Supplementary figures and legends 10](#_Toc150547539)

[Figure S1 10](#_Toc150547540)

[Figure S2 12](#_Toc150547541)

[Figure S3 13](#_Toc150547542)

[Figure S4 15](#_Toc150547543)

[Figure S5 16](#_Toc150547544)

[Figure S6 18](#_Toc150547545)

[Figure S7 19](#_Toc150547546)

[Figure S8 20](#_Toc150547547)

[Figure S9 23](#_Toc150547548)

[Figure S10 25](#_Toc150547549)

[Figure S11 26](#_Toc150547550)

[Supplementary tables 27](#_Toc150547551)

[Table S1. Clinical characteristics of the healthy individuals and hepatic hemangioma patients included in this study 27](#_Toc150547552)

[Table S2. Clinical characteristics of the liver fibrotic/cirrhotic patients included in this study 28](#_Toc150547553)

[Table S3. Primer sequences for quantitative real-time PCR analysis 29](#_Toc150547554)

[Reference 31](#_Toc150547555)

# Supplementary materials and methods

## Animal studies

All animals were housed at the Sun Yat-sen University Animal Centre and maintained under a standard 12-hour light-dark cycle. They were provided ad libitum access to food and water and remained in the study until they reached 8 weeks of age (20-25 g). The mice were randomly assigned to their respective groups, and all experiments were conducted in a blinded manner to minimize bias in treatments, genetic background, and sample collection. Genotyping was performed on the animals using polymerase chain reaction (PCR) with genomic DNA as the template, following the protocol provided on the Jackson Laboratory website (<https://mice.jax.org/>). Liver fibrosis was induced in wild-type (WT) and genotype mice using different methods. One method involved intraperitoneal injection of 20% carbon tetrachloride (CCl_4_) (diluted in olive oil) at a dose of 5 ml/kg body weight, twice per week for 8 weeks. The control group received intraperitoneal injection of olive oil at the same volume. Another method involved feeding the animals a diet containing 0.1% 3,5-diethoxycarbonyl-1,4-dihydrocollidine (DDC) for a duration of 4 weeks. *αSMA*-thymidine kinase (*αSMA*-TK) mice treated with CCl_4_ or DDC were also administered ganciclovir (GCV) at a dose of 25 mg/kg, five times a week for 8 weeks or 4 weeks, respectively. Additionally, to investigate the relationship between Periostin and proliferative aHSCs, CCl_4_ or DDC-treated *αSMA*-TK mice were simultaneously administered GCV treatment, followed by a tail vein injection of recombinant Periostin-His tagged protein (rPeriostin) (25 μg/kg, three times a week) during the latter half of the experiment. To investigate the effects of Bmp-1 in liver fibrosis, *αSMA*-TK or *Periostin* knockout (*Periostin* KO) mice were administered tail vein injections of recombinant Bmp-1-His tagged protein (rBmp-1) (25 μg/kg, three times a week) during the latter half of the CCl_4_ or DDC-induced fibrosis model. The anti-fibrotic effects of dabrafenib in liver fibrosis were investigated by simultaneously administering GCV treatment (25 mg/kg, five times a week) to CCl_4_ or DDC-treated mice.

## Histological staining

Liver tissues from humans and mice were fixed with 4% paraformaldehyde and cut into 4-µm sections. Sirius red and Hematoxylin-esoin staining were performed to detect histological injury, inflammatory infiltration, collagen deposition. For immunofluorescence in tissue samples, the slides were incubated with primary antibodies against α-SMA (Abcam, ab5694), Ki-67 (CST, 9449; Affinity, AF0198),

Collagen-I (Col-I, Abcam, ab233080), Albumin (Proteintech, 66051; Proteintech, 16475), F4/80 (CST, 70076s), Periostin (Abcam, ab79946; Adipogen, AG-20B-0033-C100), His Tag (Proteintech, 66005; Proteintech, 10001-0-AP), E-cadherin (E-cad, CST, 14472s), and N-cadherin (N-cad, CST, 14215). The semi-quantitative analysis of the histological staining was performed using Image-Pro Plus 7.0.

## Measurement of serum liver function indices.

The alanine aminotransferase (ALT) and aspartate aminotransferase (AST) levels were measured in serum samples of mice from indicated groups by using an automatic biochemical analyser (Department of Clinical Laboratory, Third Affiliated Hospital of Sun Yat-Sen University, China).

## Isolation of primary hepatocytes and HSCs

Primary hepatocytes and HSCs were isolated from the specified mice via the portal vein following anesthesia, using the previously described method(1, 2). The liver was successively perfused in situ with Ca2^+^-free HBSS (Hank’s Balanced Salt solution) for 15 minutes, followed by 100 mL of 0.2% pronase solution, and finally with 0.2% collagenase type IV until the liver appeared digested and pale in color. The obtained cell suspension was filtered through a 100 μm pore size mesh nylon filter, followed by centrifugation at 50 × g. The pellet containing primary hepatocytes was collected, while the supernatant was collected for the subsequent isolation of primary HSCs using density gradient centrifugation. Primary hepatocytes and HSCs were cultured in RPMI medium 1640 supplemented with 10% heat-inactivated fetal bovine serum.

## Cell culture and transfection

All of cell lines, including primary mouse HSCs, human HSC cell line LX-2, rat HSC cell line HSCs-T6, primary mouse hepatocytes, human HSC cell line LO-2, human hepatocyte cell line LO-2, and human monocyte cell line THP-1, were cultured in DMEM supplemented with 10% fetal bovine serum and other supplements according to the manufacturer's instructions. STR identification was performed on used cells.

Primary HSCs and LX-2 were transfected with *Periostin*-overexpressing plasmid to overexpress Periostin. Stable transfection was selected with respective antibiotics for 2 weeks and genes expression were identified successfully. The *Periostin*-overexpressing plasmid with an *HA* tag (which carries inserts encoding *Periostin*-*HA*) was cloned into the *pcDNA3.1* vector, and the lentiviral vector pLenti-*Periostin-HA* was also transfected as the control group. To evaluate the effect of Bmp-1 in hepatocytes, LO-2 and mouse primary hepatocytes were stimulated with rBmp-1 (10 μM). For inhibition of the EGFR, AZD9291 (10 μM) was added to specific hepatocytes wells. To evaluate the effect of rPeriostin on HSCs, LX-2 and mouse primary HSCs were treated with rPeriostin (10 μM) for 24 h. For inhibition of Notch-1, DAPT was added to HSC wells. To evaluate the effect of dabrafenib, *Periostin*-overexpressing mouse primary HSCs and rBmp-1-treated mouse primary hepatocytes were stimulated with dabrafenib (10 μM) for 24 h.

TGF-β-treated primary mouse HSCs, HSC-T6 cells, and LX-2 cells were seeded into 96-well plates and incubated at 37°C with 5% CO2 for 0 to 48 hours. Afterwards, 20 μl of CCK-8 (DOJINDO, CK04) solution was added to 180 μl of culture medium. Cell proliferation was assessed by measuring the absorbance at a wavelength of 450 nm after 3 hours at 37°C.

## Co-culture of HSCs with hepatocytes

Primary HSCs were isolated from mice with control or liver fibrosis mice with different genotypes and seeded 50, 000 per transwell insert (Corning, 3460) for overnight culture. After adherence, the insert was placed on the top of primary hepatocytes (100, 000 cells per well) isolated from uninjured WT mice, and for 24 h co-culture. Similarly, vector- or *Periostin*-overexpressing lentivirus-transfected LX-2 cells were co-cultured with LO-2 cells for 24 h, 48 h, and 72h in a 0.4 μm transwell plates (Corning, 3460). To evaluate the role of Bmp-1 in the crosstalk between HSCs and hepatocytes, *Periostin*-overexpressing lentivirus-transfected LX-2 cells were pre-treated with Bmp-1 inhibitor (UK-383367, 10 μM) for 12 h. Subsequently, they were co-cultured with LO-2 cells. Primary hepatocytes and LO-2 cells were stimulated with rBmp-1 (10 μM) for 12 h. Additionally, primary hepatocytes that had undergone co-culture with primary HSCs were stimulated with rBmp-1 (10 μM). Primary hepatocytes and LO-2 lysate were harvested for further experiments, and cells were also fixed with 4% paraformaldehyde for staining.

## RNA extraction and quantitative real-time PCR

The RNAgents Total RNA Isolation System (Promega, Madison, WI, USA) was used to isolate total RNA and then transcribed into cDNA using a High Capacity cDNA Kit (TOYOBO, FSQ101) according to the manufacturer’s instruction. A Chromo 4 Detector System (MJ Research, Sierra Point, CA, USA) was performed to detect the expression of the indicated mRNA using gene specific primers. Each gene expression was normalized by *βactin* from the same sample and presented as fold changes relative to the matched control values. Primer sequences are listed in Table. S3.

## RNA sequencing

The total RNA from mouse liver was isolated using TRIzol reagent following standard protocols. After quality inspection with the Agilent 2100 Bioanalyzer (Agilent, cat. G2939AA, CA, USA) and NanoPhotometer® (Implen, cat. N60, Munich, Germany), poly(A) mRNA was purified from 1 μg total RNA using VAHTS® mRNA Capture Beads with Oligo (dT) (Vazyme, cat. N401-01, Nanjing, China) through two rounds of purification. Subsequently, mRNA fragments were fragmented using the VAHTS® Universal V6 RNA-seq Library Prep Kit (Vazyme, cat. NR604, Nanjing, China), followed by reverse transcription into cDNA, which was used to synthesize U-labeled second-stranded DNAs. RNA sequencing was performed on the isolated RNA using a HiSeq 2000 (Illumina, San Diego, CA). Raw data was obtained as FASTQ files, and concentration and fragment size were assessed using the Agilent 2100 Bioanalyzer (Agilent, cat. G2939AA, CA, USA) and Qubit assay tubes (Life, cat. 1604220, CA, USA). The expression level of mRNA was calculated using RSEM (RNA-Seq by Expectation Maximization) (v1.3.1) and normalized to FPKM (Fragments Per Kilobase Per Million reads). The R package "limma" was utilized for analysis and screening of differentially expressed genes. A log2 Fold Change of ≥ 1 and a P-Value < 0.05 were considered statistically significant.

## Tandem Mass Tag (TMT) quantitative nanoLC-MS/MS proteomics

The cell supernatant was collected from LX-2 cells treated with vehicle or rPeriostin. RIPA lysate was added, from which protein was extracted and TMT labeling was performed according to the Thermo labeling kit instructions. For each sample, 200 ng of total peptides were separated and analyzed with a nano-UPLC (nanoElute2) coupled to a timsTOF Pro2 instrument (Bruker) with a nano-electrospray ionsource. Separation was performed using a reversed-phase column (PePSep C18, 1.9 𝜇, 75𝜇 × 15 cm, Bruker, Germany). The mass spectrometer adopts DDA PaSEF mode for DDA data acquisition, and the scanning range is from 100 to 1700 m/z for MS1. MS spectra lists were searched against their species-level UniProt FASTA databases (uniprot_Homo sapiens_9606_reviewed_2023_09. fasta), with Carbamidomethyl [C] , TMT 6 plex(K), TMT 6 plex (N-term)as a fixed modification and Oxidation (M) and Acetyl (Protein N-term) as variable modifications. The false discovery rate (FDR) was set to 0.01 for both PSM and peptide levels. Peptide identification was performed with an initial precursor mass deviation of up to 10 ppm and a fragment mass deviation of 0.02Da. Unique peptide and Razor peptide were used for protein quantification and total peptide amount for normalization. All the other parameters were reserved as default.

## Western blotting analysis

Liver tissue protein was extracted from tissue lysates for western blotting. Denatured proteins were separated on Tris-glycine polyacrylamide gels by SDS-PAGE and transferred to PVDF membranes. The PVDF membranes were incubated at 4 °C with primary antibodies against Cyclin E1 (Abcam, ab33911), PCNA (Abcam, ab29), α-SMA (Abcam, ab5694), Col-I (Abcam, ab233080), Collagen-IV (Col- IV, Abcam, ab6586), Periostin (Abcam, ab79946; Adipogen, AG-20B-0033-C100), E-cad (CST, 14472s), N-cad (N-cad, CST, 14215), Vimentin (Vim, CST, 5741), Bmp-1 (Abcam, ab205394), and anti-β-actin (Abcam, ab8226). Then, corresponding horseradish peroxidase-conjugated secondary antibodies were used to identify the primary antibody/antigen complexes. Finally, membrane-bound antibodies were detected by a hypersensitive chemiluminescence detection reagent.

## Construction of recombinant protein

The purity of the recombinant Periostin protein with C-terminal His Tag exceeded 95% and was verified by PAGE analysis. For practical application, the protein was reconstituted in a diluent containing 20 mM Tris and 150 mM NaCl at pH 8.0. The recombinant Bmp-1 protein with N-terminal His Tag was expressed in *E. coli* and then lyophilized from a Tris-based buffer solution containing 50% Glycerol for preservation and subsequent use.

## Enzyme-linked immunosorbent assay (ELISA)

The Human Periostin ELISA kit (FineTest, EH0255) was used to examine the Periostin levels in human serum from healthy individual and patients with liver fibrosis. Analyses were performed according to the kit’s instructions.

## Data collection and analysis

We collected single-cell RNA sequencing (scRNA-seq) and bulk RNA sequencing datasets from humans and mice (GSE171904, GSE136103, GSE48452, GSE15654, and GSE136103) through the Gene Expression Omnibus (GEO) database. To analyze and integrate the read matrix, perform clustering, and conduct differential gene expression analyses, we utilized the R package “Seurat”. We applied a threshold of unique counts, filtering out cell doublets with counts exceeding 5,000 or less than 500. Normalization for each cell was performed using the “LogNormalize” method based on the total expression. The “FindVariableGenes” function with default settings was used to identify highly variable genes across the single cells. The top 100 markers were selected to generate an expression heatmap of the marker genes. For survival analysis, we employed the R packages “survival” and “survminer” to plot Kaplan-Meier curves. Box plots were generated using the R package “ggplot2”, and beeswarm plots were generated using the R package “ggbeeswarm”.

# Supplementary figures and legends

## Figure S1





**Figure S1. Deconvolution of the liver cells in humans and mice.**

Analysis of liver single-cell RNA sequencing (scRNA-seq) dataset obtained from oil and CCl_4_-treated mice (GSE171904) (A-G):

(A) Uniform manifold approximation and projection (UMAP) visualization of cell types in liver, based on single-cell transcriptomes pooled from oil and CCl_4_-treated mice.

(B) Identification of the main cell types in mouse liver.

(C) Heatmap displaying the differentially expressed genes for each cell type in mouse liver.

(D) Analysis of normalized percentages of qHSCs and aHSCs in the control and CCl_4_ groups showed that aHSCs are predominantly found in mice with liver fibrosis.

(E-F) GO enrichment analysis of RNA-sequencing data from qHSCs and aHSCs.

(G) Violin plots showing the expression of proliferation markers *CCNE1*, *Ki-67*, and *PCNA* in non-pHSCs and pHSCs.

Analysis of liver scRNA-seq dataset obtained from normal control and liver fibrosis patients (GSE136103) (H-J):

(H) UMAP visualization of cell types in liver, based on single-cell transcriptomes pooled from normal controls and liver fibrosis patients.

(I) Identification of the main cell types in human liver.

(J) Violin plots showing the expression of *αSMA*, *Col4a1*, and *PCNA* in the indicated subsets of HSCs.

ECs, endothelial cells, qHSCs, quiescent HSCs; aHSCs, activated HSCs; pHSCs, proliferative HSCs.

## Figure S2


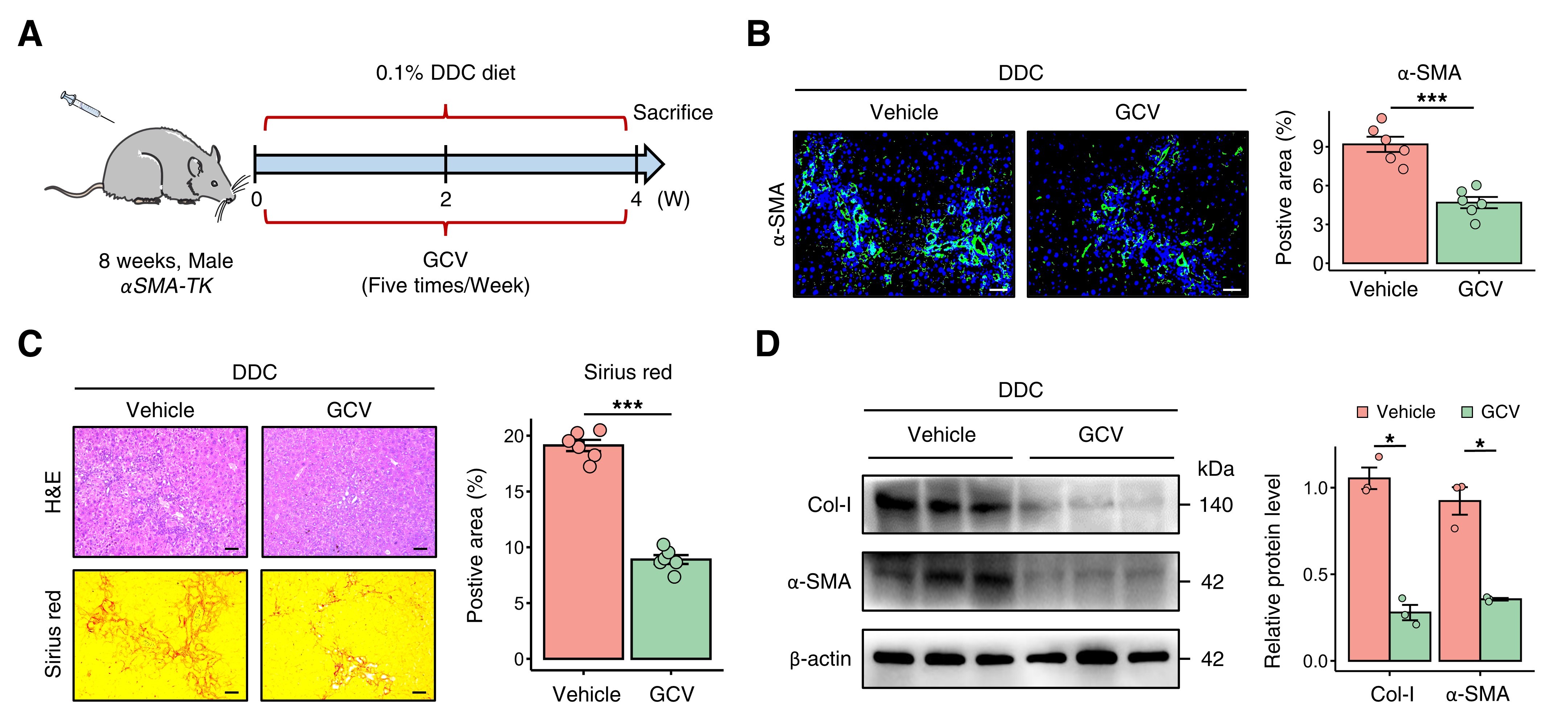


**Figure S2.** **Depletion of proliferative HSCs attenuates liver fibrosis in DDC administrated-mice.**

(A) Ganciclovir (GCV)-administration strategy in *αSMA*-TK mice treated with DDC to evaluate the impact of proliferative aHSCs on liver fibrogenesis (n = 6 per group).

(B) Immunofluorescence staining showed a decrease in α-SMA levels in DDC-induced α*SMA*-TK mice treated with GCV. The data were quantified (n = 6 per group) (Scale bar: 50 μm).

(C) H&E and Sirius red staining in liver sections of *αSMA*-TK mice from indicated groups. The data were quantified (n = 6 per group) (Scale bar: 100 μm).

(D) After treatment with GCV, protein levels of Collagen-I (Col-I), and α-SMA were reduced in αSMA-TK mice.

All results are shown as mean ± SEM. **p* < 0.05; ****p* < 0.001. TK, thymidine kinase; DDC, 3,5-diethoxycarbonyl-1,4-dihydrocollidine.

## Figure S3


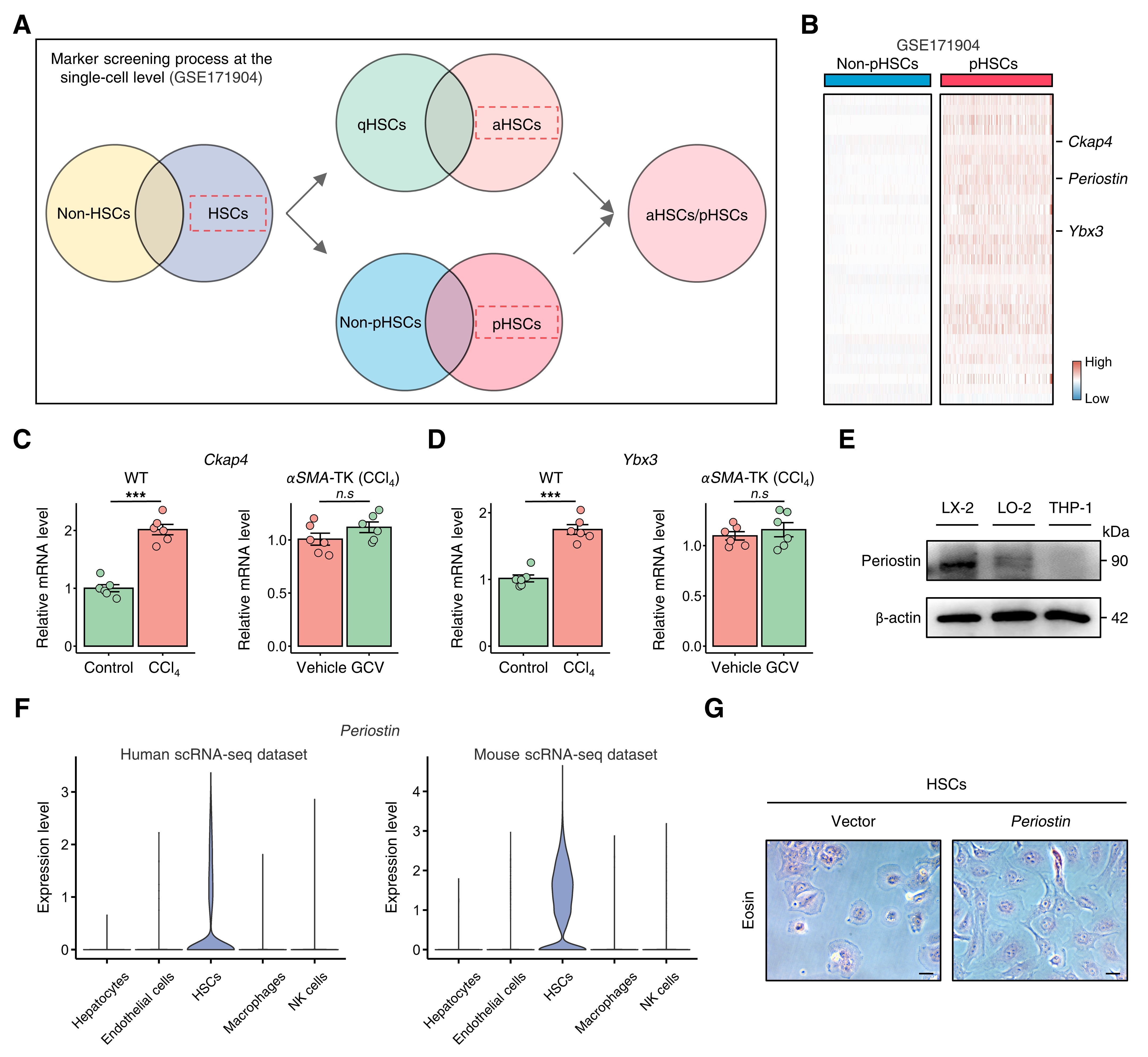


**Figure S3. Identification of marker genes for proliferative aHSCs.**

(A) Schematic overview of marker screening at the single-cell level (GSE171904): HSCs were extracted from liver cell pools and classified into qHSCs, aHSCs, and pHSCs based on known markers. Subsequently, the overlapping genes between the gene sets of aHSCs and pHSCs were screened to identify potential marker genes for the proliferative aHSCs.

(B) Heatmap depicting the expression of *Ckap4*, *Postn*, and *Ybx* (overlapping genes between the gene sets of aHSCs and pHSCs) in non-pHSCs and pHSCs.

(C-D) mRNA levels of *Ckap4* and *Ybx3* in liver tissues of WT and *αSMA*-TK mice from indicated groups.

(E) Periostin protein levels were higher in the LX-2 cells (HSCs) than in LO-2 cells (hepatocytes) and THP-1 cells (monocytes). The data were quantified (n = 3 independent experiments).

(F) Violin plots showing the expression of *Periostin* in mainly parenchyma and non-parenchyma cells from human and mouse liver scRNA-seq data.

(G) Eosin staining showed the activation-like morphology in LX-2 cells overexpression *Periostin* (Scale bar: 25 μm).

All results are shown as mean ± SEM. **p* < 0.05; ****p* < 0.001. qHSCs, quiescent HSCs; aHSCs, activated HSCs; pHSCs, proliferative HSCs; WT, wild type; TK, thymidine kinase; CCl_4_, carbon tetrachloride; GCV, ganciclovir.

## Figure S4


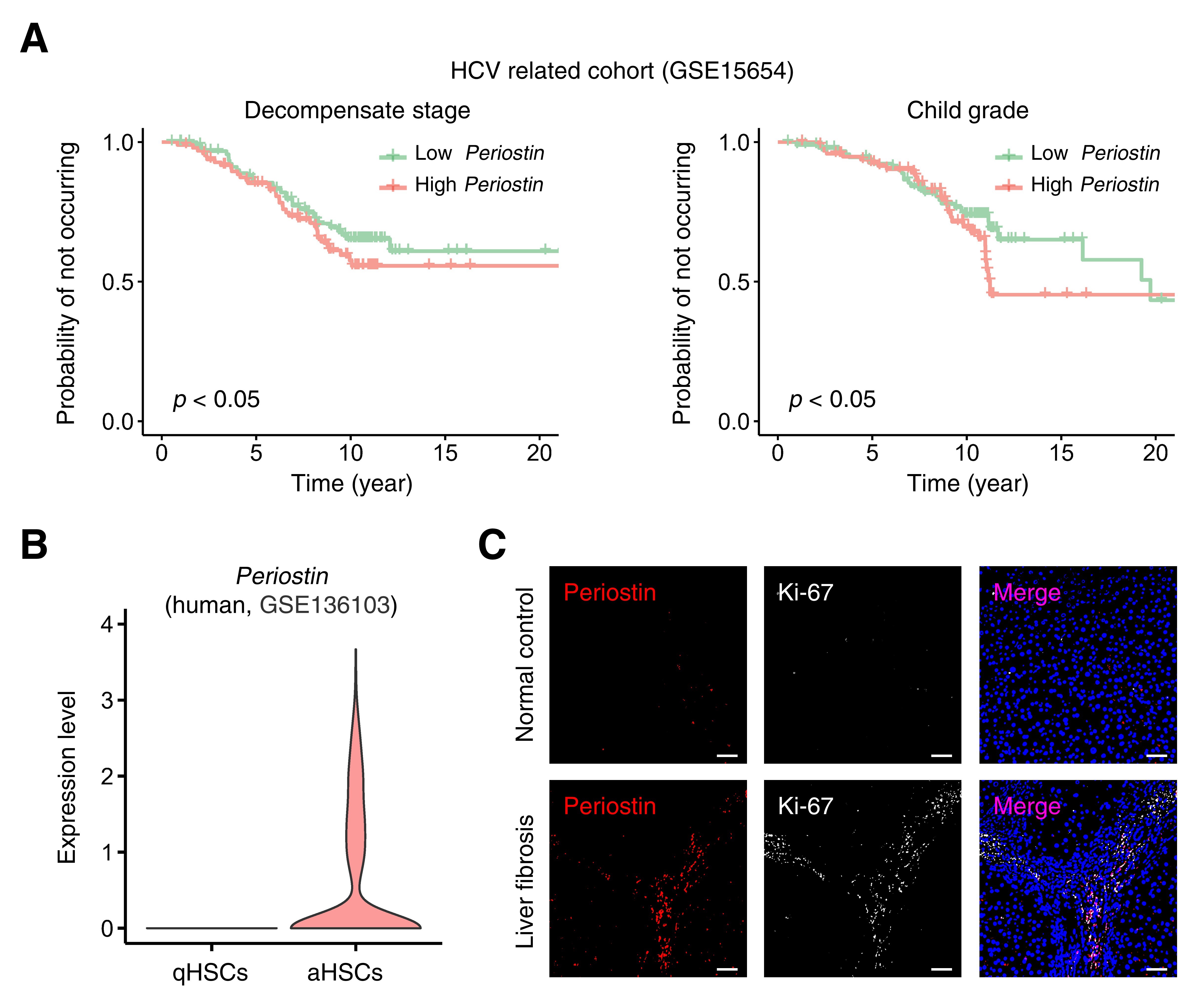


**Figure S4. *Periostin* expression and its prognostic performance in humans**

(A) HCV-infected liver fibrosis patients with high levels of *Periostin* in the liver exhibited an increased probability of developing decompensate cirrhosis stage (left) and severe Child grade (right).

(B) Violin plots showing the expression of *Periostin* in qHSCs and aHSCs derived from an scRNA-seq dataset of both normal controls and liver fibrosis patients (GSE136103).

(C) Immunofluorescence staining showed the expression and distribution of Periostin and Ki-67 in liver sections from normal controls and liver fibrosis patients (Scale bar: 50 μm).

qHSCs, quiescent HSCs; aHSCs, activated HSCs.

## Figure S5


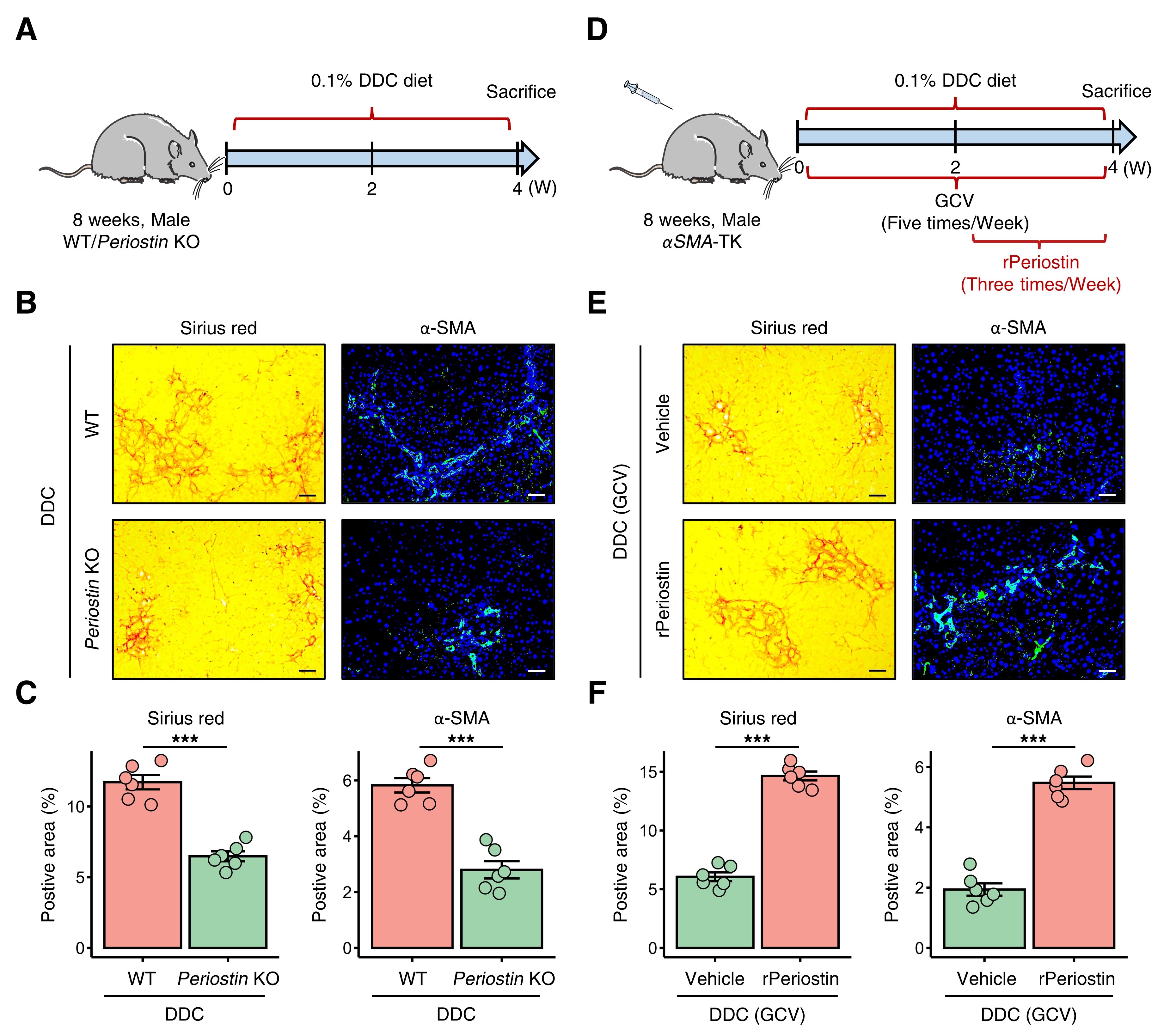


**Figure S5. Periostin is involved in the development of DDC-induced liver fibrosis in mice.**

(A) Schematic overview illustrating the construction process of the DDC-induced liver fibrosis model in WT and *Periostin* KO mice (n = 6 per group).

(B-C) Sirius red and α-SMA staining demonstrated that the absence of Periostin mitigated liver fibrosis in murine models induced by DDC. The data were quantified (n = 6 per group) (Scale bar: 50 μm).

(D) Schematic overview illustrating the experimental strategy of administering recombinant Periostin-His tagged protein (rPeriostin) in DDC-induced *αSMA*-TK mice treated with GCV (n = 6 per group).

(E-F) Sirius red and α-SMA staining in liver sections of *αSMA*-TK mice from indicated groups. The data were quantified (n = 6 per group) (Scale bar: 50 μm).

All results are shown as mean ± SEM. **p* < 0.05; ****p* < 0.001. DDC, 3,5-diethoxycarbonyl-1,4-dihydrocollidine; WT, wild type; KO, knockout; TK, thymidine kinase; GCV, ganciclovir.

## Figure S6


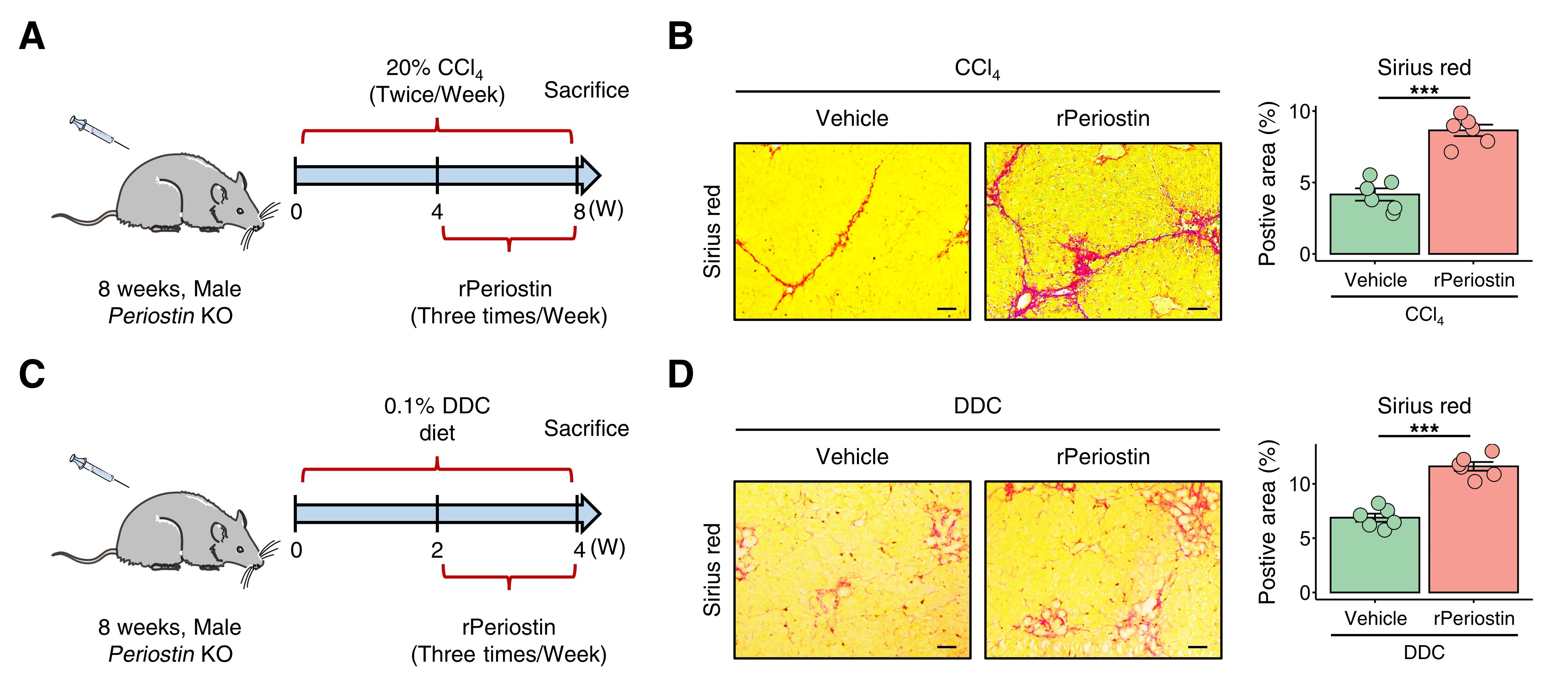


**Figure S6. Exogenous Periostin reverses the mitigation of liver fibrosis in Periostin-deficient mice.**

(A) Schematic overview depicting the administration strategy of rPeriostin in *Periostin* KO mice treated with CCl_4_ (n = 6 per group).

(B) Sirius red staining demonstrated the reversal of liver fibrosis attenuation in *Periostin* KO mice following treatment with rPeriostin. The data were quantified (n = 6 per group) (scale bar: 50 μm).

(C) Schematic overview depicting the administration strategy of rPeriostin in *Periostin* KO mice treated with DDC (n = 6 per group).

(D) Sirius red staining in liver sections of *Periostin* KO mice from the indicated groups. The data were quantified (n = 6 per group) (scale bar: 50 μm).

All results are shown as mean ± SEM. **p* < 0.05; ****p* < 0.001. KO, knockout; CCl_4_, carbon tetrachloride; DDC, 3,5-diethoxycarbonyl-1,4-dihydrocollidine; rPeriostin, recombinant Periostin-His tagged protein.

## Figure S7


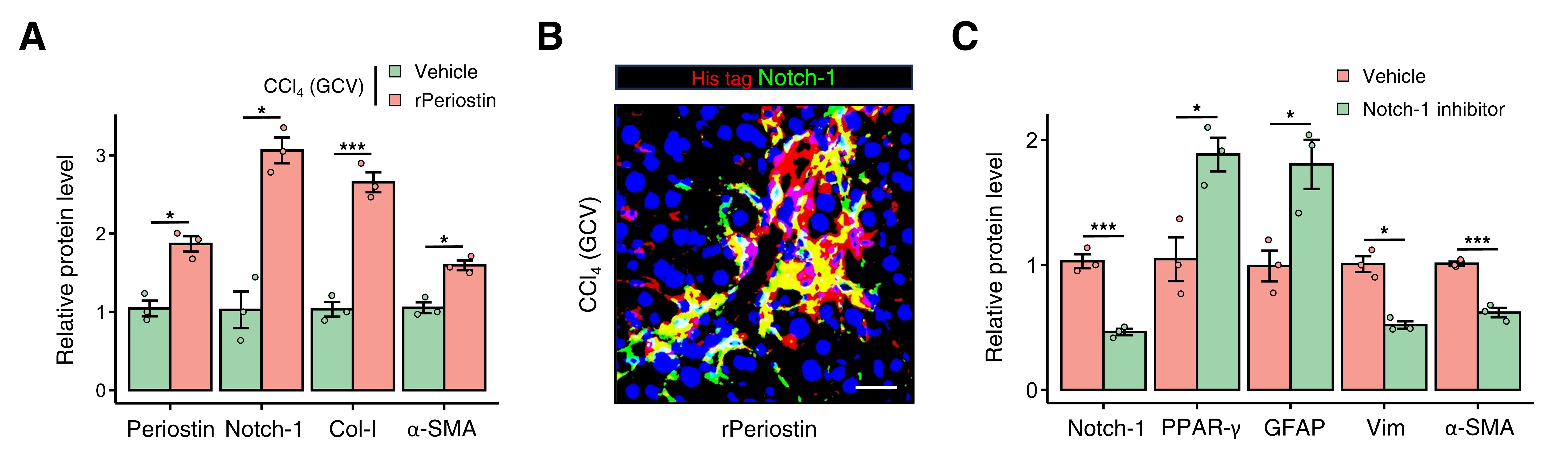


**Figure S7. Notch-1 is involved in the Periostin-induced phenotypic transition of HSCs**

(A) Quantification of protein expression levels of Periostin, Notch-1, Col-I, and α-SMA in liver tissues from *αSMA*-TK mice from the indicated groups.

(B) Immunofluorescence staining demonstrated the co-localization of rPeriostin (red) and Notch-1 (green) in the liver of α*SMA*-TK mice treated with rPeriostin (Scale bar: 25 μm).

(C) Quantification of protein expression levels of Notch-1, PPAR-γ, GFPA, Vim, and α-SMA in rPeriostin treated-primary HSCs (isolated from un-injured WT mice) with or without administration of Notch-1 inhibitor (10 μM).

All results are shown as mean ± SEM. **p* < 0.05; ****p* < 0.001. CCl_4_, carbon tetrachloride; GCV, ganciclovir; rPeriostin, recombinant Periostin-His tagged protein; Vim, Vimentin.

.

## Figure S8


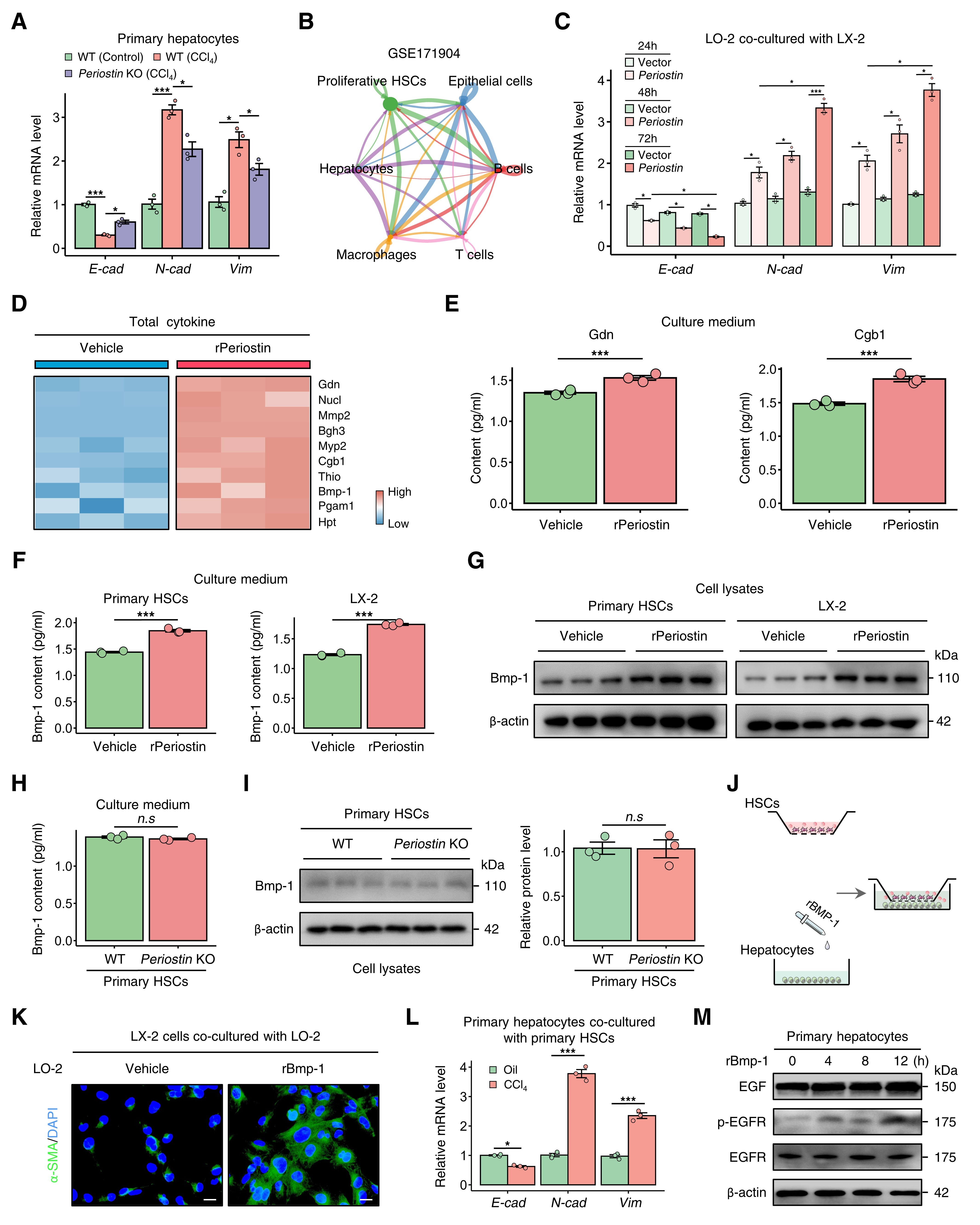


**Figure S8. Crosstalk between Periostin-expressing HSCs and hepatocytes through Bmp-1**

(A) mRNA levels of *E-cad*, *N-cad*, and *Vim* in primary hepatocytes isolated from WT and *Periostin* KO mice treated with or without CCl_4_.

(B) Cellchat analysis was performed on scRNA-seq data from mouse liver (GSE171904).

(C) The mRNA levels of *E-cad* were downregulated, while *N-cad* and *Vim* were upregulated in LO-2 cells co-cultured with *Periostin*-overexpressing LX-2 cells, at 24h, 48h, and 72 hours.

(D) Whole protein profile analysis of culture medium from LX-2 cells treated with or without rPeriostin.

(E) ELISA showing the Gdn and Cgb1 content in the culture medium of LX-2 cells treated with or without rPeriostin (10 μM).

(F) ELISA showing the Bmp-1 content in the culture medium of primary HSCs (isolated from un-injured mice) and LX-2 cells treated with or without rPeriostin (10 μM).

(G) Protein expression levels of Bmp-1 in primary HSCs (isolated from un-injured mice) and LX-2 cells under different treatment conditions.

(H) There was no statistically significant difference between Bmp-1 content in the culture medium of primary HSCs isolated from WT and *Periostin* KO mice.

(I) Protein expression levels of Bmp-1 in primary HSCs from WT and *periostin* KO mice. The data were quantified (n = 3 per group).

(J) Schematic diagram illustrating the co-culture of rBmp-1-treated hepatocytes with HSCs.

(K) The levels of α-SMA were unregulated in LX-2 co-cultured with rBmp-1-treated LO-2 (Scale bar: 25 μm).

(L) mRNA levels of *E-cad*, *N-cad*, and *Vim* in primary hepatocytes (isolated from un-injured mice) co-cultured with primary HSCs isolated from oil or CCl_4_-induced mice.

(M) Protein expression levels of EGF, p-EGFR, and EGFR in primary hepatocytes treated rBmp-1 (10 μM) at 0, 4 h, 8 h, and 12 h (n = 3 independent experiments).

All results are shown as mean ± SEM. **p* < 0.05; ****p* < 0.001. WT, wild type; KO, knockout; CCl_4_, carbon tetrachloride; pHSCs, proliferative HSCs; rPeriostin, recombinant Periostin-His tagged protein; rBmp-1, recombinant rBmp-1-His tagged protein; E-cad, E-cadherin; N-cad, N-cadherin; Vim, Vimentin.

## Figure S9


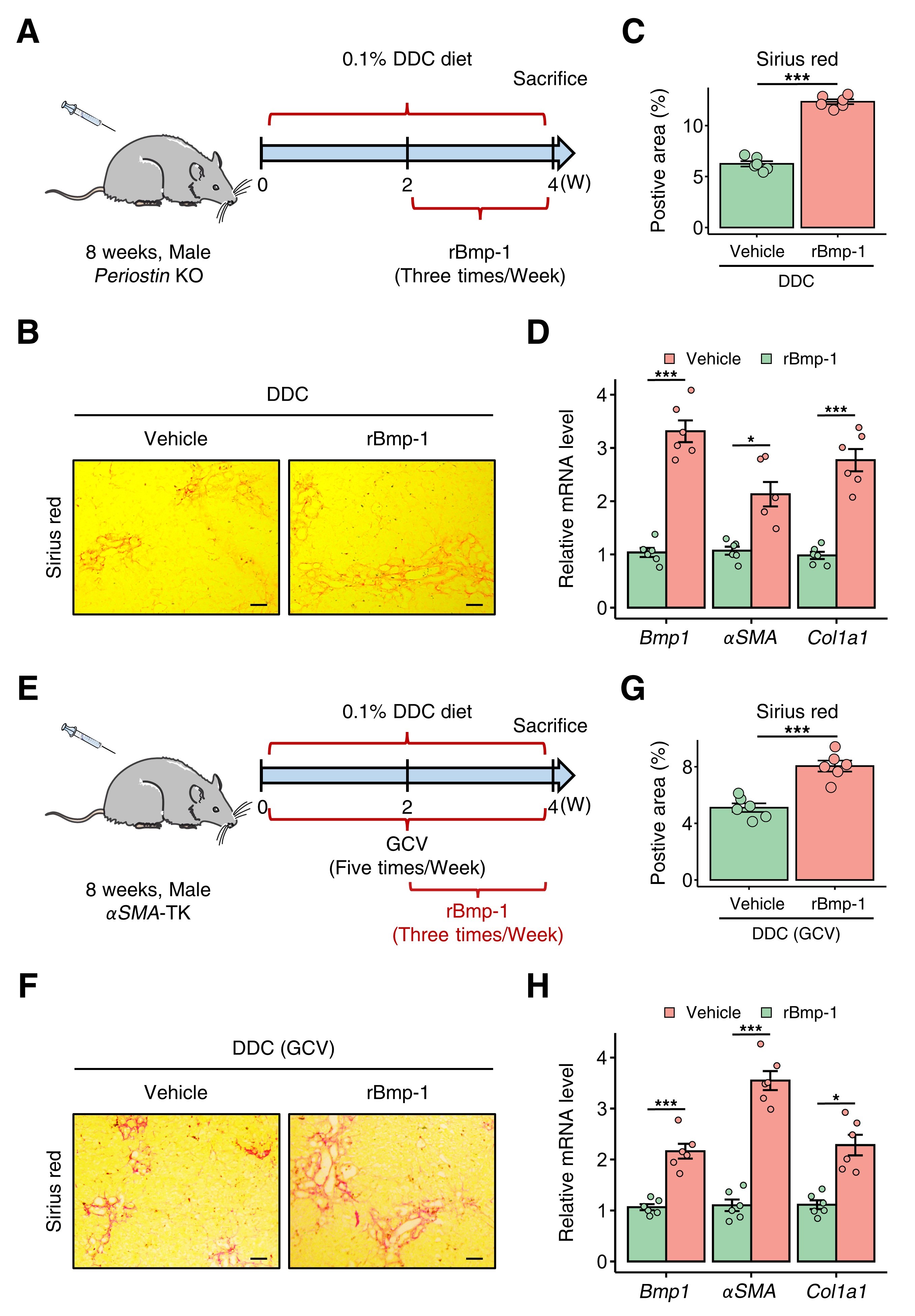


**Figure S9. Bmp-1 plays a crucial role in the progression of liver fibrosis induced by DDC administration in mice.**

(A) Schematic overview depicting the administration strategy of rBmp-1 in *Periostin* KO mice treated with DDC (n = 6 per group).

(B-C) Sirius red staining demonstrated the reversal of liver fibrosis attenuation in *Periostin* KO mice following treatment with rBmp-1. The data were quantified (n = 6 per group) (Scale bar: 50 μm).

(D) qPCR analysis demonstrated that treatment with rBmp-1 significantly upregulated the levels of *Bmp1*, *αSMA*, and *Col1a1* in DDC-induced *Periostin* KO mice.

(E) Schematic overview illustrating the experimental strategy of administering rBmp-1 in DDC-induced *αSMA*-TK mice treated with GCV (n = 6 per group).

(F-G) Sirius red staining in liver sections of *αSMA*-TK mice from indicated groups. The data were quantified (n = 6 per group) (Scale bar: 50 μm).

(H) The mRNA levels of *Bmp1*, *αSMA*, and *Col1a1* were upregulated in liver tissues of *αSMA*-TK mice, following rBmp-1 treatment.

All results are shown as mean ± SEM. **p* < 0.05; ****p* < 0.001. KO, knockout; DDC, 3,5-diethoxycarbonyl-1,4-dihydrocollidine; rBmp-1, recombinant rBmp-1-His tagged protein; TK, thymidine kinase; GCV, ganciclovir.

## Figure S10


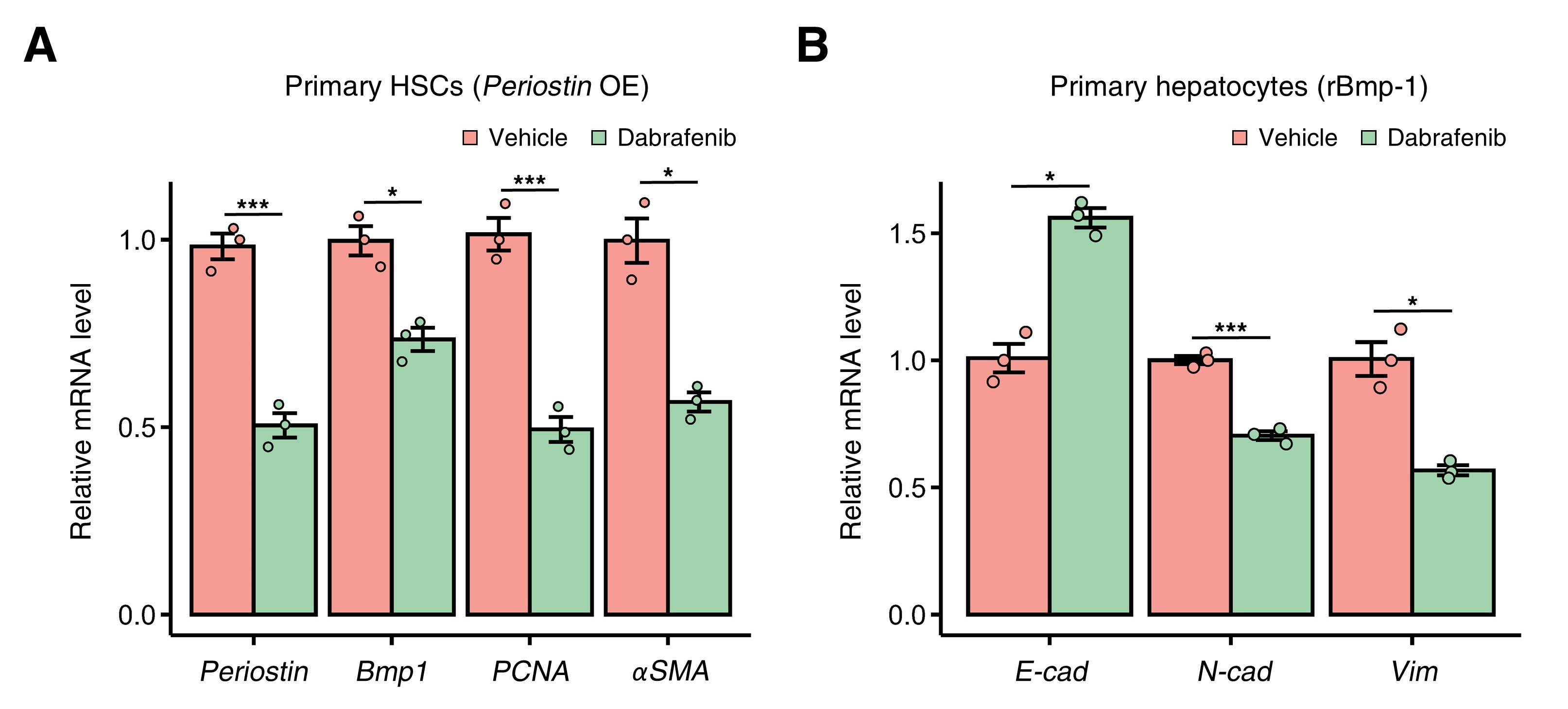


**Figure S10. Antifibrotic effects of dabrafenib *in vitro* experiments**

(A) mRNA levels of *Periostin*, *Bmp1*, *PCNA*, and *αSMA* in *Periostin*-overexpressing primary HSCs (isolated from un-injured mice) treated with or without dabrafenib.

(B) mRNA levels of *E-cad*, *N-cad*, and *Vim* in rBmp-1-treated primary hepatocytes (isolated from un-injured mice) in the indicated groups.

All results are shown as mean ± SEM. **p* < 0.05; ****p* < 0.001. OE, overexpression; rBmp-1, recombinant Bmp-1-His tagged protein; E-cad, E-cadherin; N-cad, N-cadherin; Vim, Vimentin.

## Figure S11


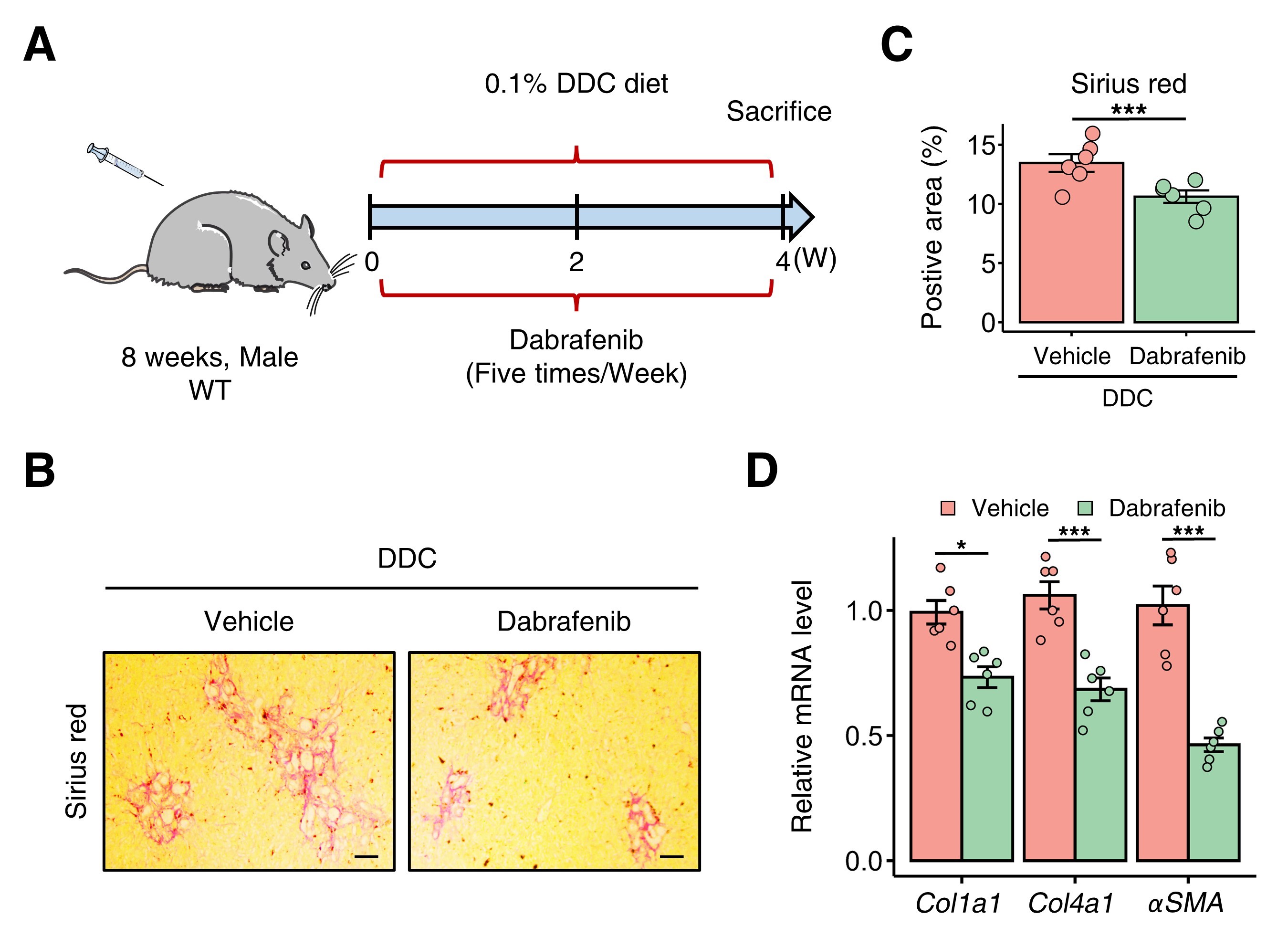


**Figure S11. Dabrafenib** **alleviates liver fibrosis in murine models induced by DDC by targeting Periostin**

(A) Schematic overview of the experimental setup for assessing the efficacy of dabrafenib in a DDC-induced liver fibrosis mouse model (n = 6 per group).

(B-C) Dabrafenib treatment in DDC-induced mice resulted in reduced liver fibrosis, as indicated Sirius red staining. The data were quantified (n = 6 per group) (Scale bar: 50 μm).

(D) mRNA levels of *Col1a1*, *Col4a1*, and *αSMA* in liver tissues of mice treated with or without dabrafenib

All results are shown as mean ± SEM. **p* < 0.05; ****p* < 0.001. WT, wild type; DDC, 3,5-diethoxycarbonyl-1,4-dihydrocollidine.

# Supplementary tables

## Table S1. Clinical characteristics of the healthy individuals and hepatic hemangioma patients included in this study

| **NO.** | **Gender (male/female)** | **Age (years)** | **Hepatitis** | **Ishak Score** |
| --- | --- | --- | --- | --- |
| 1 | male | 52 | - | 0 |
| 2 | female | 56 | - | 0 |
| 3 | female | 46 | - | 0 |
| 4 | male | 43 | - | 0 |
| 5 | male | 55 | - | 0 |
| 6 | male | 50 | - | 0 |
| 7 | female | 52 | - | 0 |
| 8 | male | 51 | - | 0 |
| 9 | female | 60 | - | 0 |
| 10 | male | 47 | - | 0 |
| 11 | female | 47 | - | 0 |
| 12 | male | 47 | - | 0 |
| 13 | male | 54 | - | 0 |
| 14 | female | 51 | - | 0 |
| 15 | female | 44 | - | 0 |
| 16 | male | 41 | - | 0 |
| 17 | male | 43 | - | 0 |
| 18 | male | 49 | - | 0 |
| 19 | male | 48 | - | 0 |
| 20 | female | 52 | - | 0 |

## Table S2. Clinical characteristics of the patients with liver fibrosis.

| **NO.** | **Gender (male/female)** | **Age (years)** | **Ascites** | **Hepatic encephalopathy** | **Child-Pugh Score** | **Ishak Score** |
| --- | --- | --- | --- | --- | --- | --- |
| 1 | female | 46 | + | - | A | 1 |
| 2 | male | 48 | + | + | C | 4 |
| 3 | male | 57 | + | + | B | 3 |
| 4 | male | 51 | + | - | B | 2 |
| 5 | female | 65 | + | - | C | 4 |
| 6 | male | 51 | + | - | C | 2 |
| 7 | female | 67 | + | + | C | 3 |
| 8 | male | 71 | + | + | C | 4 |
| 9 | male | 42 | - | - | A | 2 |
| 10 | female | 54 | - | - | B | 2 |
| 11 | female | 57 | + | - | B | 3 |
| 12 | male | 57 | + | - | C | 3 |
| 13 | female | 62 | + | - | C | 3 |
| 14 | male | 61 | + | + | C | 4 |
| 15 | male | 49 | - | - | A | 1 |
| 16 | female | 56 | + | - | C | 4 |
| 17 | male | 57 | - | - | A | 2 |
| 18 | male | 63 | + | + | C | 4 |
| 19 | male | 45 | - | - | B | 2 |
| 20 | male | 54 | - | - | A | 1 |

## Table S3. Primer sequences for quantitative real-time PCR analysis

| **Name** | **Sequence (5’- 3’)** |
| --- | --- |
| m-*αSMA*-F | GTCCCAGACATCAGGGAGTAA |
| m-*αSMA*-R | TCGGATACTTCAGCGTCAGGA |
| m-*CCNE1*-F | GTGGCTCCGACCTTTCAGTC |
| m-*CCNE1*-R | CACAGTCTTGTCAATCTTGGCA |
| m-*Ki67*-F | AGCACAAAGAGACGGTCTAAGA |
| m-*Ki67*-R | CTCTGCCTCGTGACTGTGTT |
| m-*PCNA*-F | TTTGAGGCACGCCTGATCC |
| m-*PCNA*-R | GGAGACGTGAGACGAGTCCAT |
| m-*Col1a1*-F | GGGGCAAGACAGTCATCGAA |
| m-*Col1a1*-R | GGGTGGAGGGAGTTTACACG |
| m-*Col4a1*-F | CTGGAGAAAAGGGCCAGAT |
| m-*Col4a1*-R | TCCTTAACTTGTGCCTGTCCA |
| m-*Periostin*-F | CCTGCCCTTATATGCTCTGCT |
| m-*Periostin*-R | AAACATGGTCAATAGGCATCACT |
| m-*E-cadherin*-F | ACTGTGAAGGGACGGTCAAC |
| m-*E-cadherin*-R | GGAGCAGCAGGATCAGAATC |
| m-*N-cadherin*-F | GGCAGAAGAGAGACTGGGTC |
| m-*N-cadherin*-R | GAGGCTGGTCAGCTCCTGGC |
| m-*Vimentin*-F | ACTCACCTGTGAAGTGGATGC |
| m-*Vimentin*-R | TGGTATTCACGAAGGTGACG |
| m-*PPARγ*-F | TCGCTGATGCACTGCCTATG |
| m-*PPARγ*-R | GAGAGGTCCACAGAGCTGATT |
| m-*GFAP*-F | GGGGCAAAAGCACCAAAGAAG |
| m- *GFAP*-R | GGGACAACTTGTATTGTGAGCC |
| m-*Bmp1*-F | TTGTACGCGAGAACATACAGC |
| m-*Bmp1*-R | CTGAGTCGGGTCCTTTGGC |
| m-*Ckap4*-F | TCCCGTCAGAGGGATGAGC |
| m-*Ckap4*-R | GCTGGGAGTTTCTCAGGAGG |
| m-*Ybx3*-F | TCCTGTAGAAGGGAGTCGCTA |
| m- *Ybx3*-R | CACCAGCATTACGGGGAGG |
| m-*βactin*-F | GGCTGTATTCCCCTCCATCG |
| m-*βactin*-R | CCAGTTGGTAACAATGCCATGT |
| h-*αSMA*-F | TTCAATGTCCCAGCCATGTA |
| h-*αSMA*-R | GAAGGAATAGCCACGCTCAG |
| h-*CCNE1*-F | GCCAGCCTTGGGACAATAATG |
| h-*CCNE1*-R | CTTGCACGTTGAGTTTGGGT |
| h-*Ki67*-F | ACGCCTGGTTACTATCAAAAGG |
| h-*Ki67*-R | CAGACCCATTTACTTGTGTTGGA |
| h-*PCNA*-F | CCTGCTGGGATATTAGCTCCA |
| h-*PCNA*-R | CAGCGGTAGGTGTCGAAGC |
| h-*Periostin-*F | CTCATAGTCGTATCAGGGGTCG |
| h- *Periostin*-R | ACACAGTCGTTTTCTGTCCAC |
| h-*E-cadherin*-F | ATTTTTCCCTCGACACCCGAT |
| h-*E-cadherin*-R | TCCCAGGCGTAGACCAAGA |
| h-*N-cadherin*-F | TCAGGCGTCTGTAGAGGCTT |
| h-*N-cadherin*-R | ATGCACATCCTTCGATAAGACTG |
| h-*Vimentin*-F | AGTCCACTGAGTACCGGAGAC |
| h-*Vimentin*-R | CATTTCACGCATCTGGCGTTC |
| h-*PPARγ*-F | GGGATCAGCTCCGTGGATCT |
| h-*PPARγ*-R | TGCACTTTGGTACTCTTGAAGTT |
| h-*GFAP*-F | AGGTCCATGTGGAGCTTGAC |
| h-*GFAP*-R | GCCATTGCCTCATACTGCGT |
| h-*βactin*-F | GTCTTCCCCTCCATCGTG |
| h-*βactin*-R | AGGGTGAGGATGCCTCTCTT |

**Reference**

1. Tan S, Liu H, Ke B, Jiang J, Wu B. The peripheral CB(1) receptor antagonist JD5037 attenuates liver fibrosis via a CB(1) receptor/beta-arrestin1/Akt pathway. Br J Pharmacol. 2020;177(12):2830-2847.

2. Tan S, Liu X, Chen L, Wu X, Tao L, Pan X, et al. Fas/FasL mediates NF-kappaBp65/PUMA-modulated hepatocytes apoptosis via autophagy to drive liver fibrosis. Cell Death Dis. 2021;12(5):474.
