# Supplementary material for: Activated hepatic stellate cell-derived Bmp-1 induces liver fibrosis via mediating hepatocyte epithelial-mesenchymal transition
Source: Cell Death Dis. 2024 Jan 12;15(1):41. doi: 10.1038/s41419-024-06437-8 (PMC10786946; doi:10.1038/s41419-024-06437-8)

Figure 1J

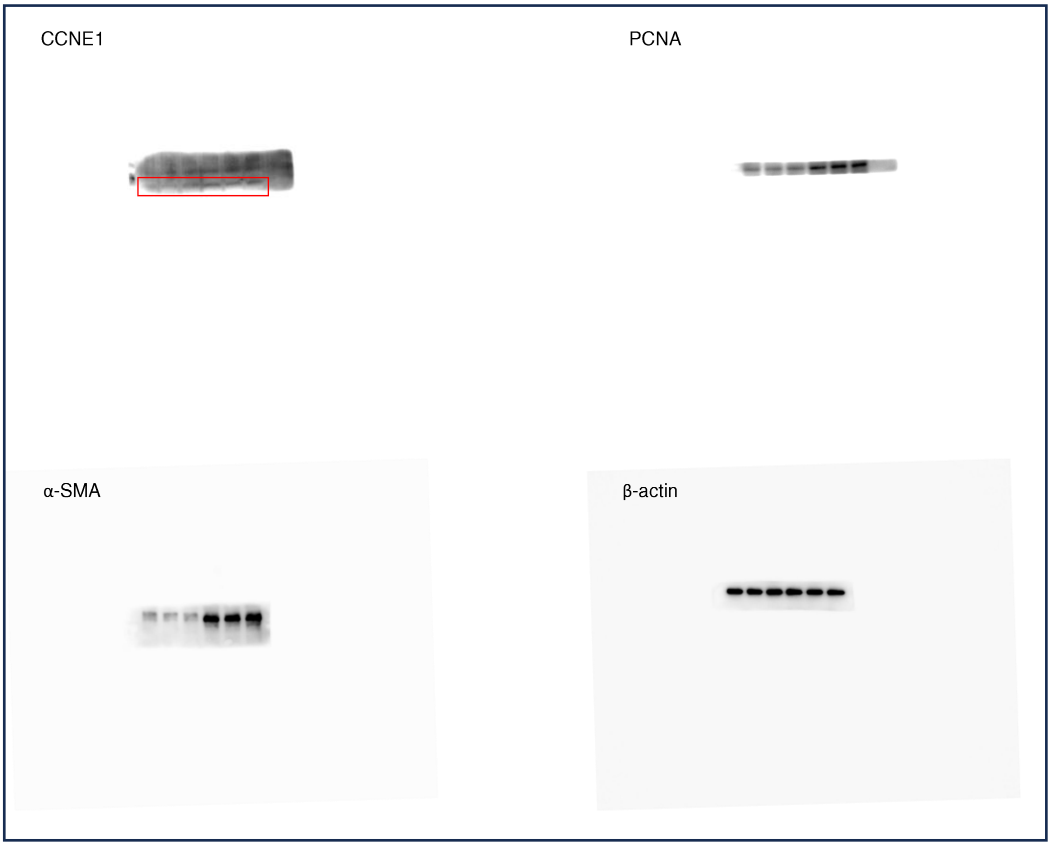

Figure 1P

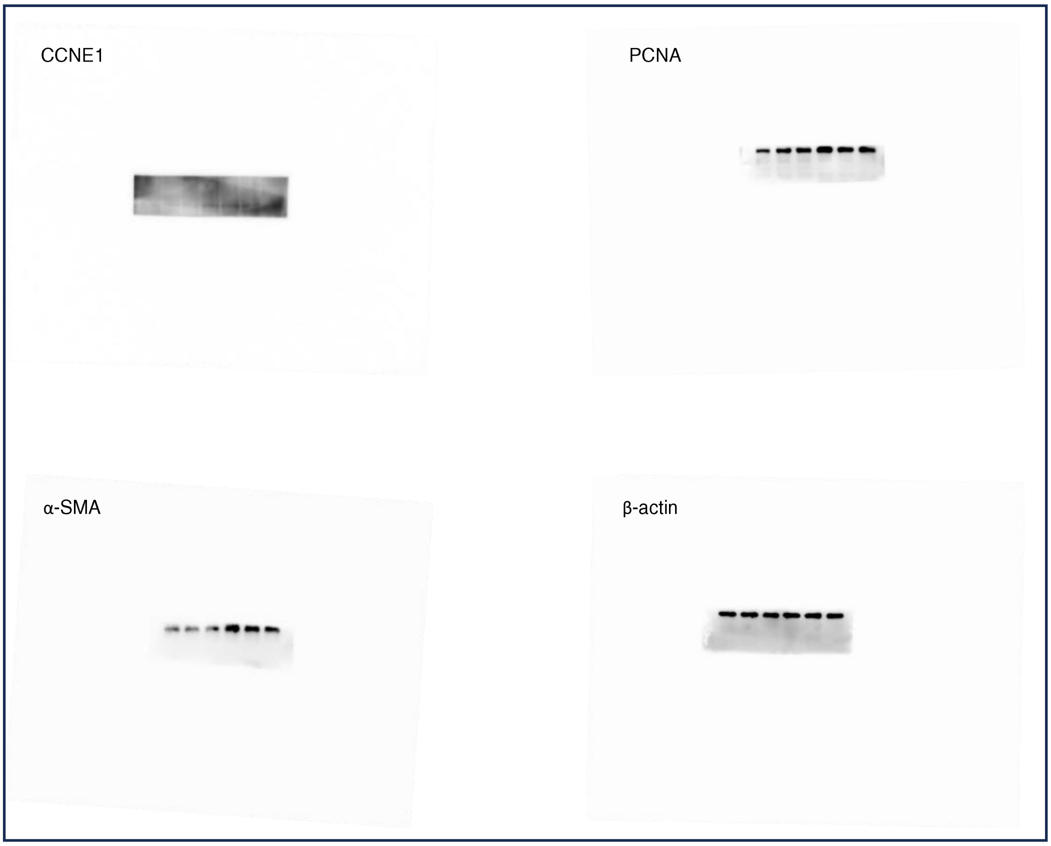

Figure 2C

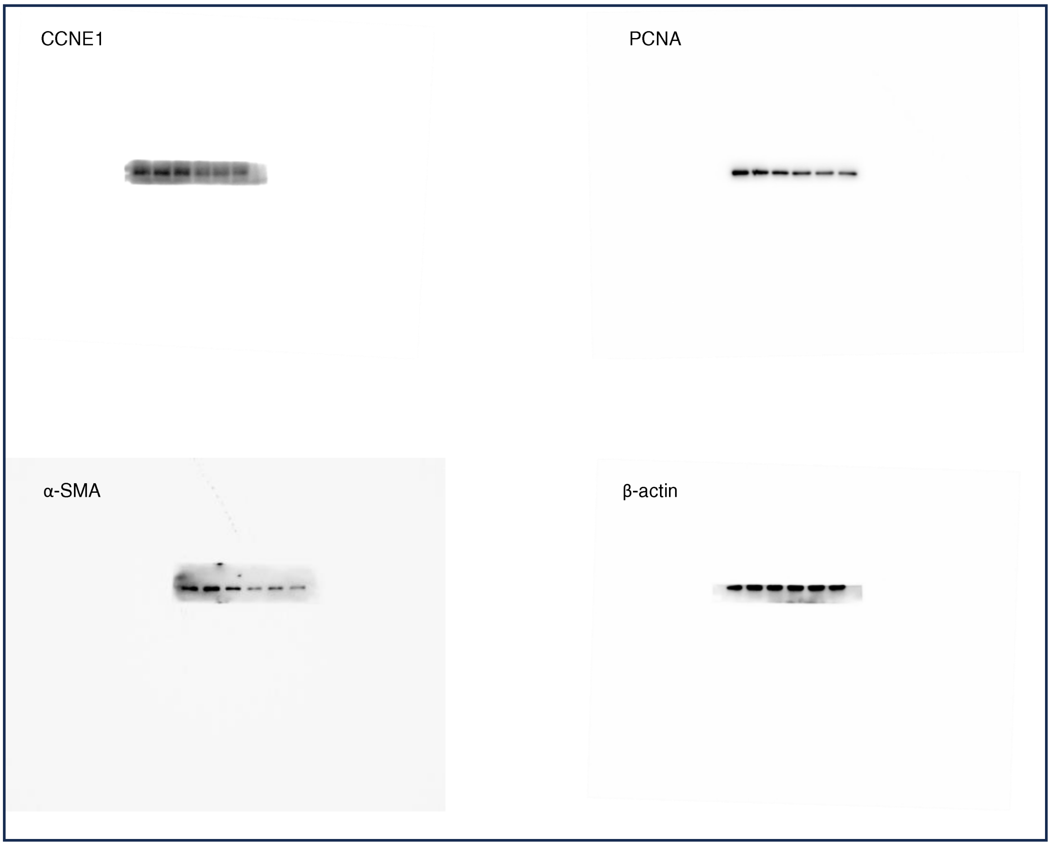

Figure 2G

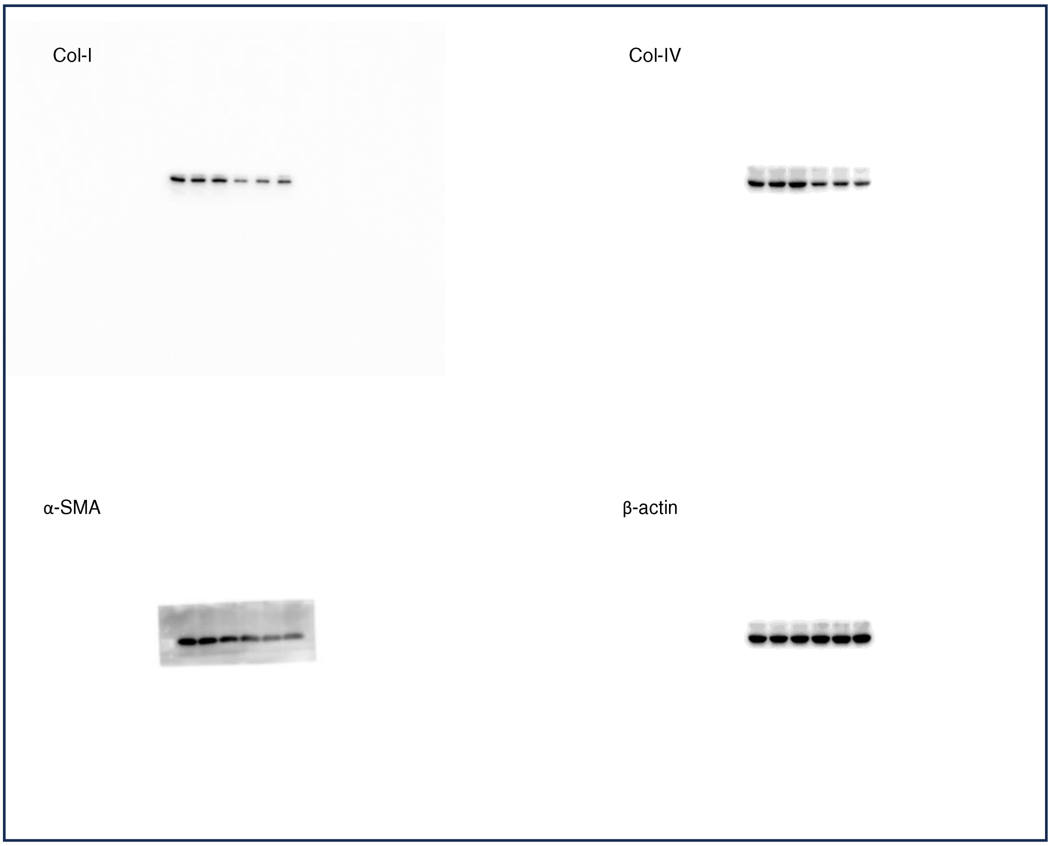

Figure 3E Left

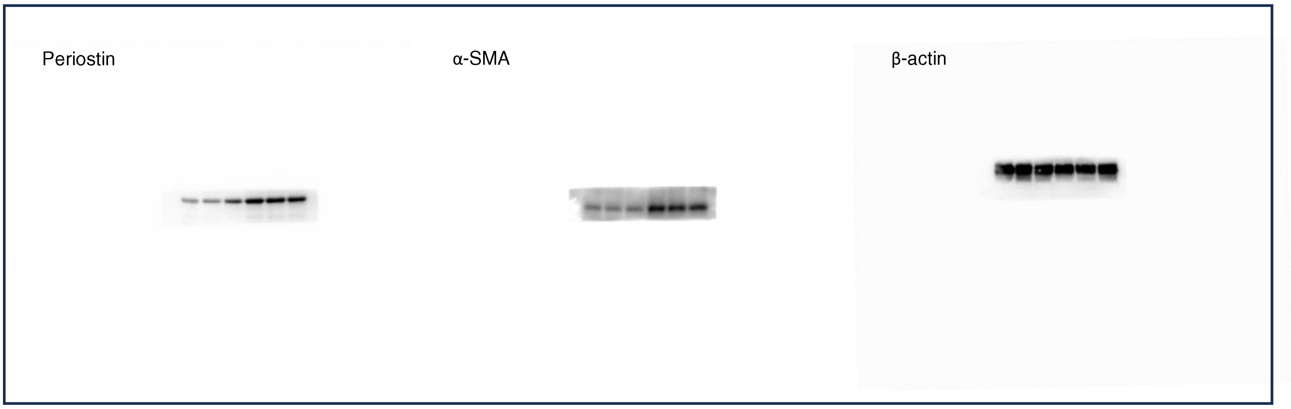

Figure 3E Right

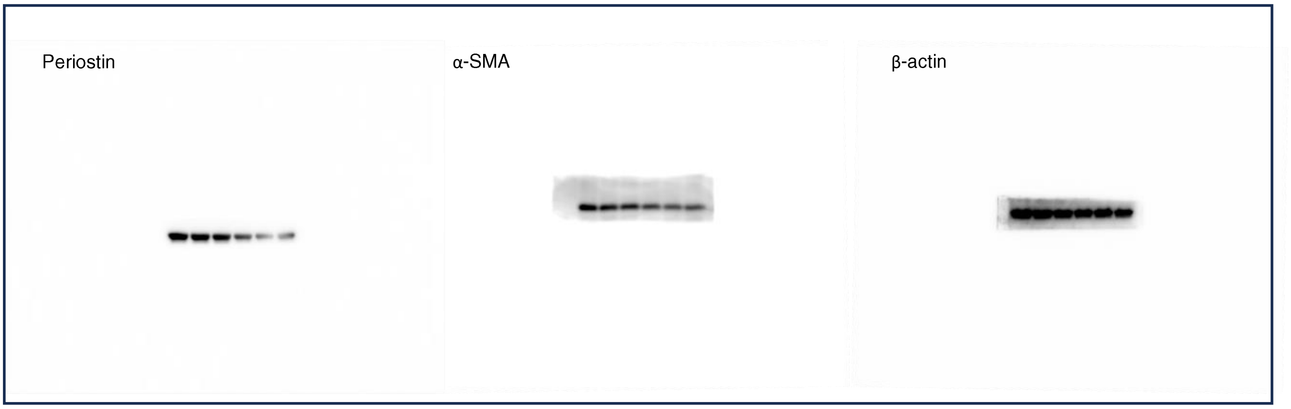

Figure 3H Left

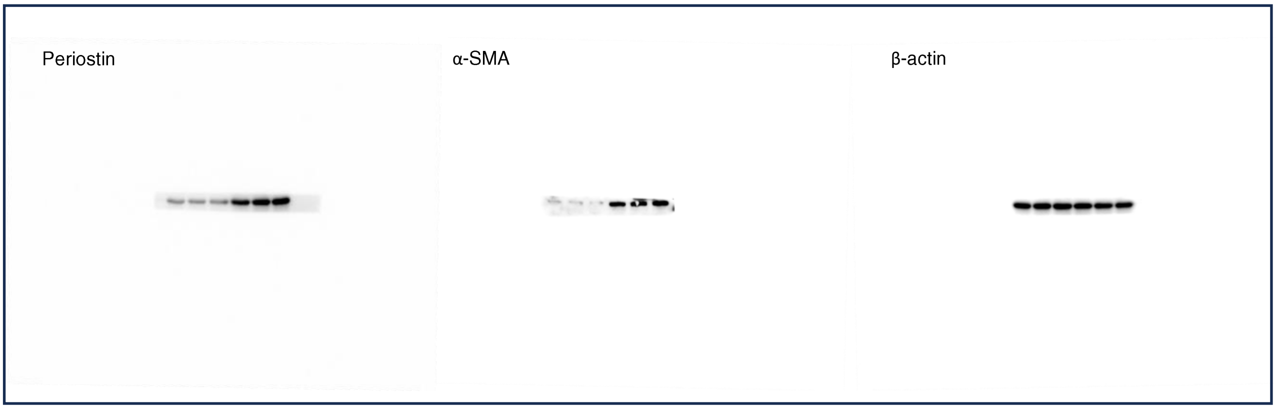

Figure 3H Mid

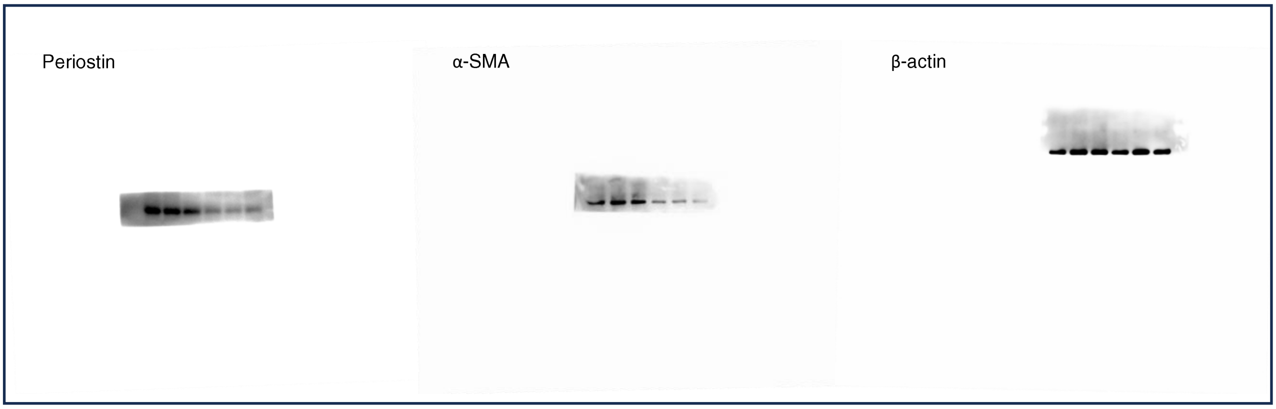

Figure 3H Right

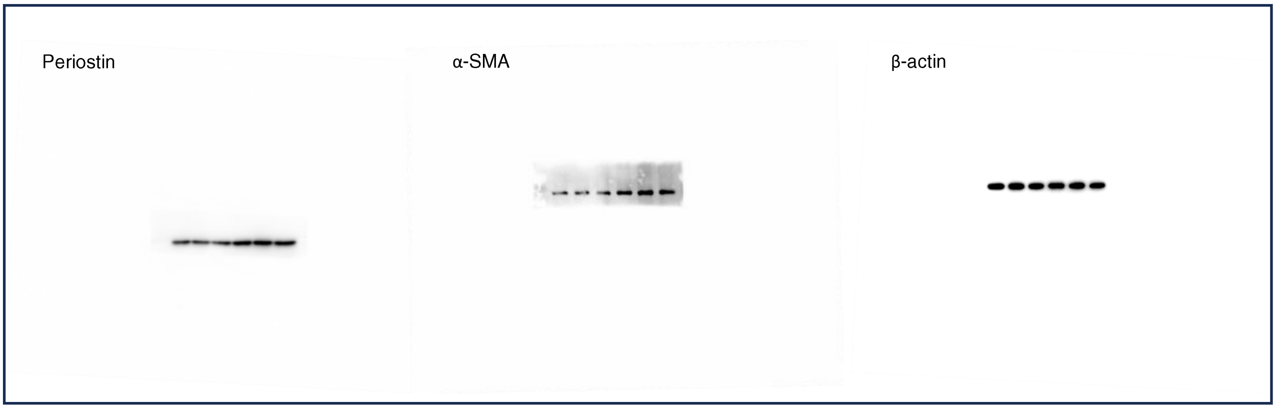

Figure 3J Left

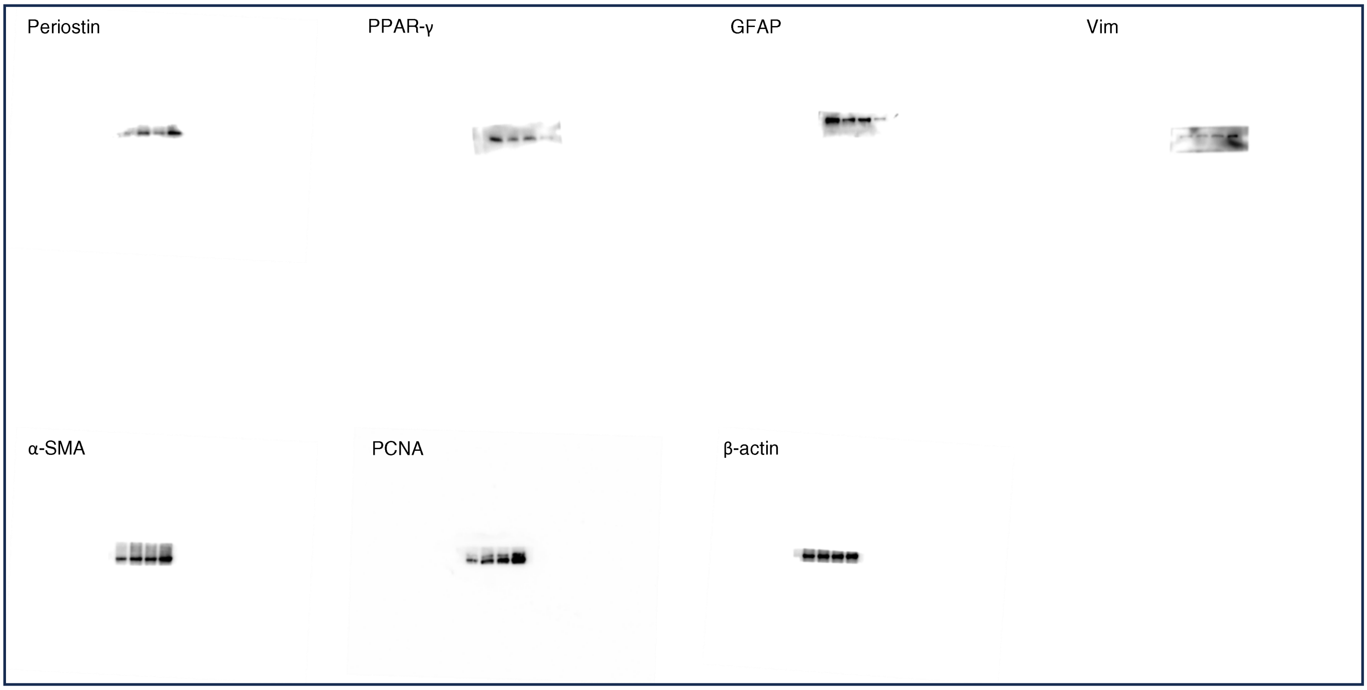

Figure 3J Right

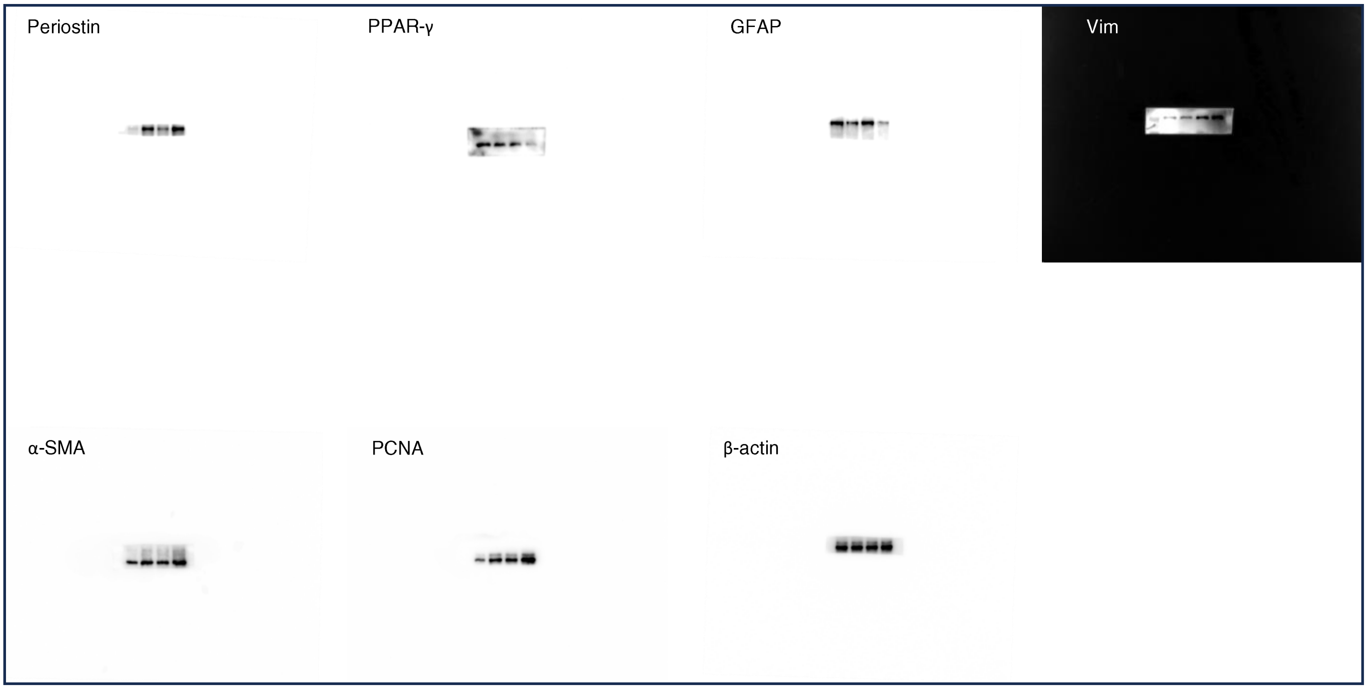

Figure 4E

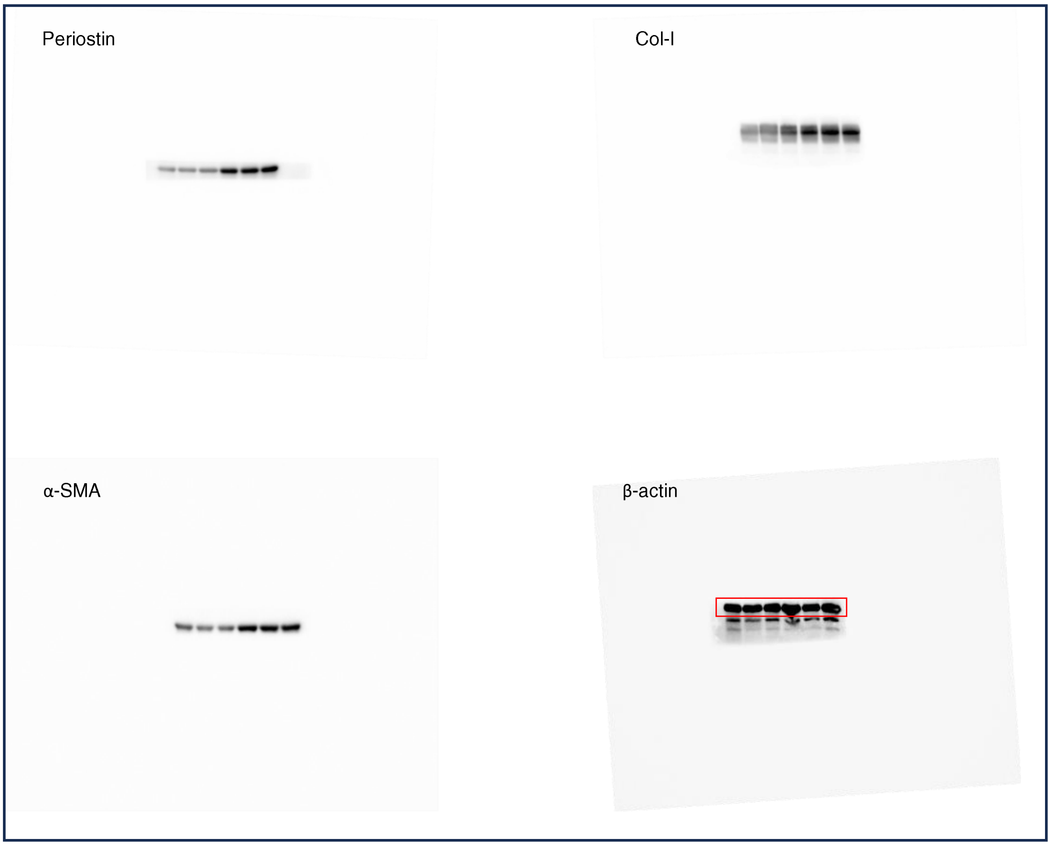

Figure 5C

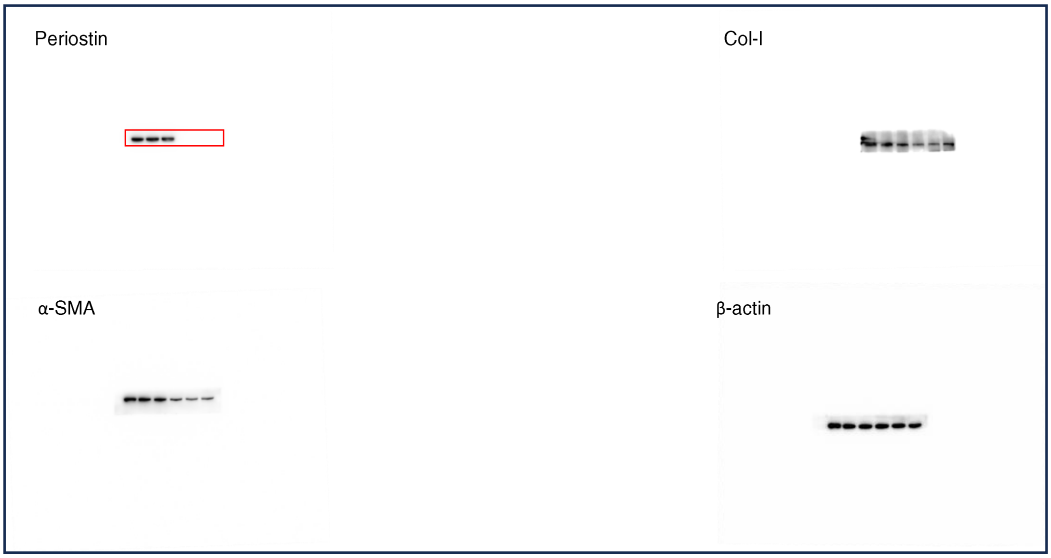

Figure 5E

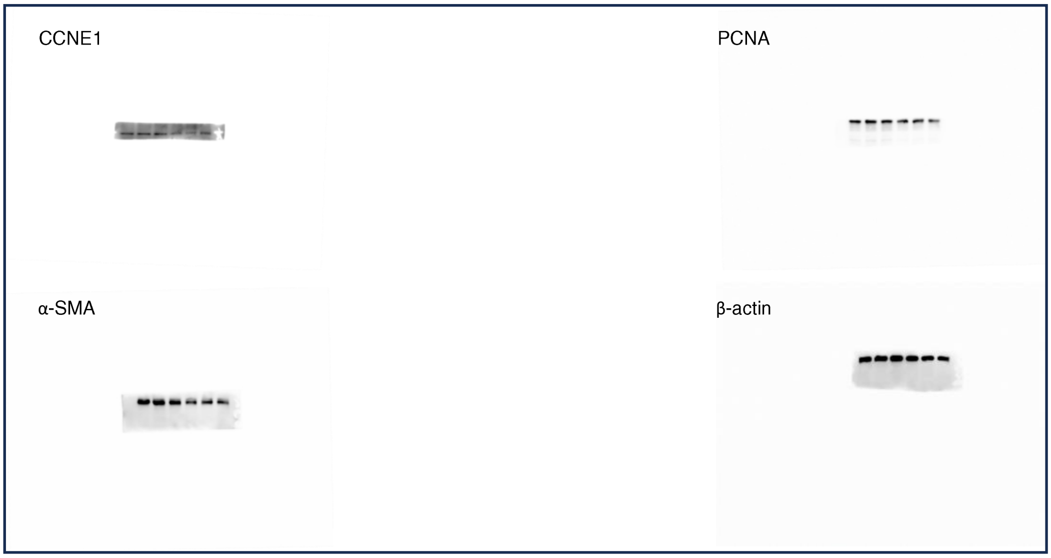

Figure 5J

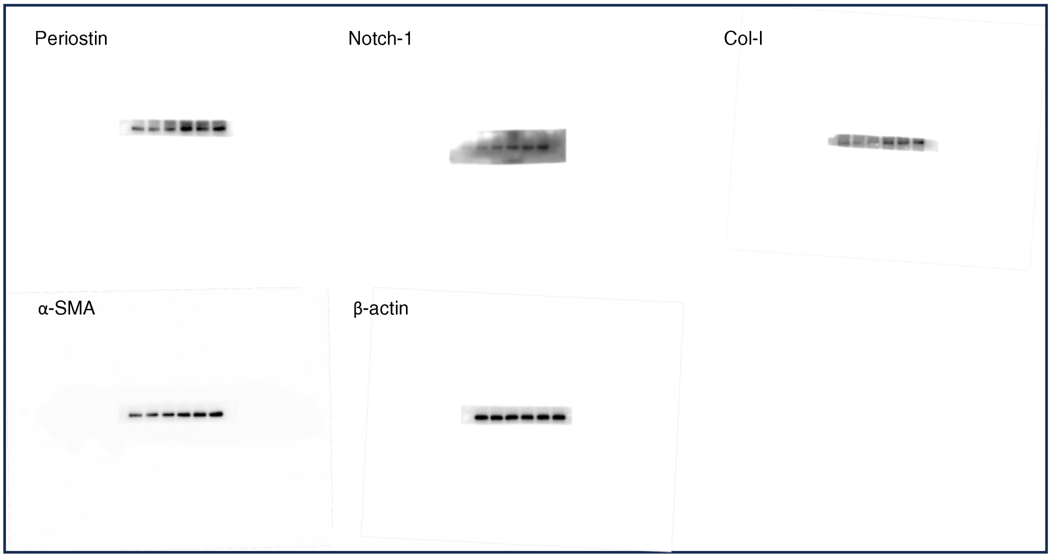

Figure 5K

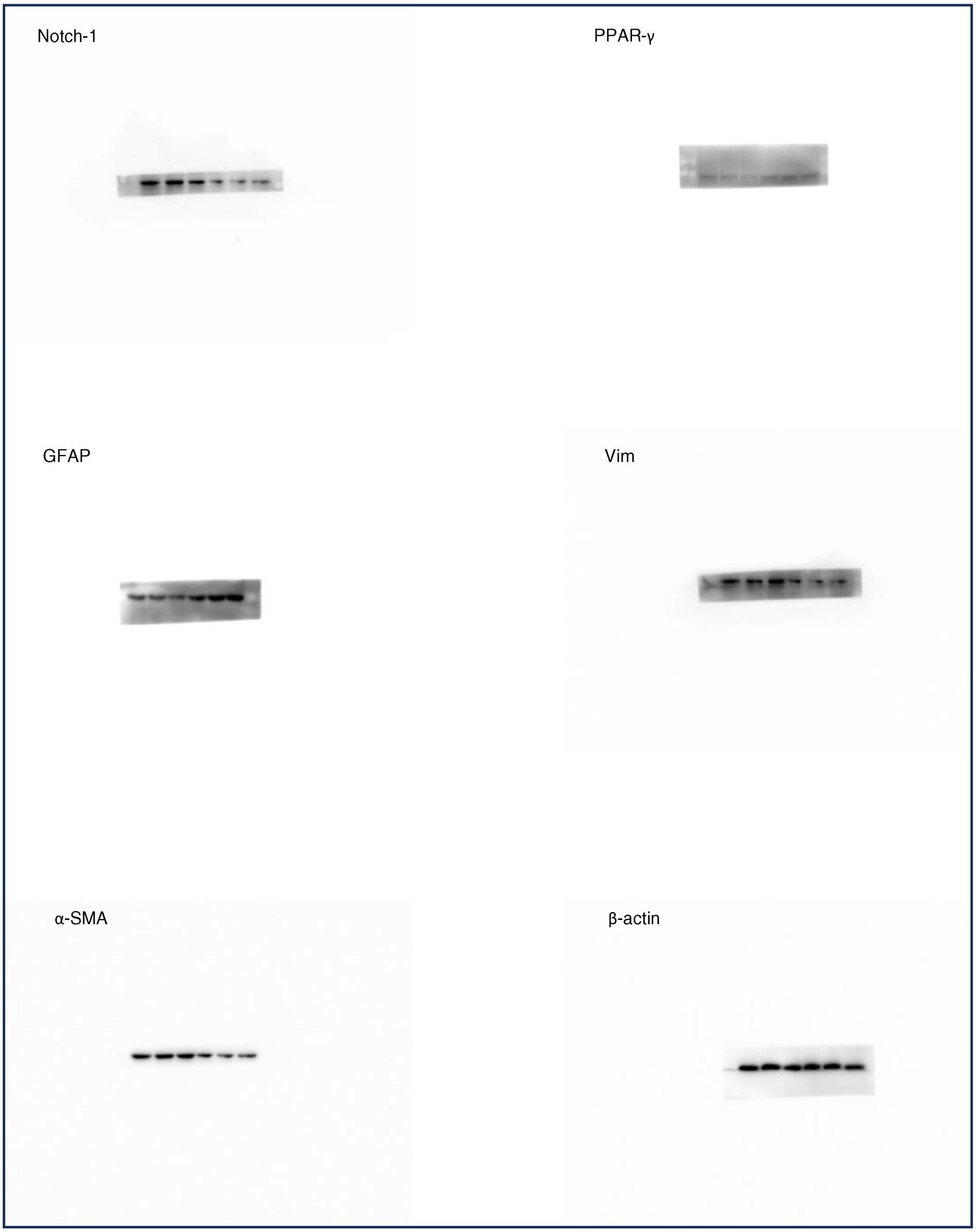

Figure 6F

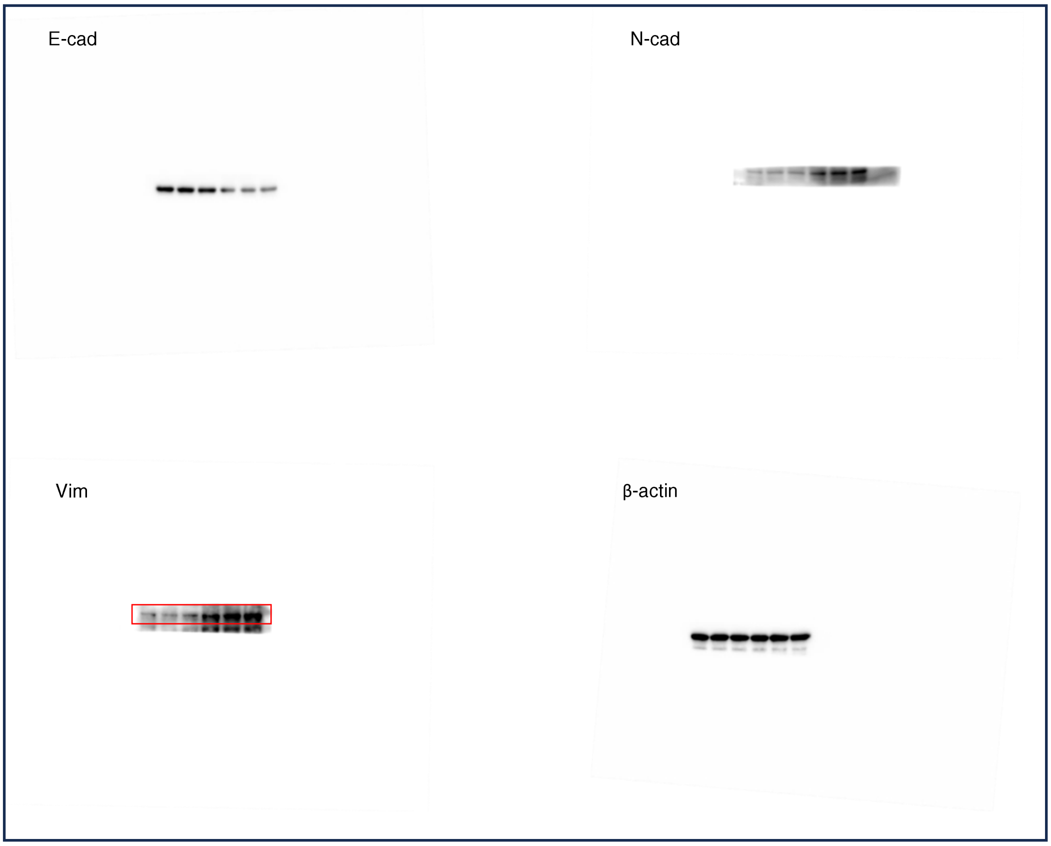

Figure 6J

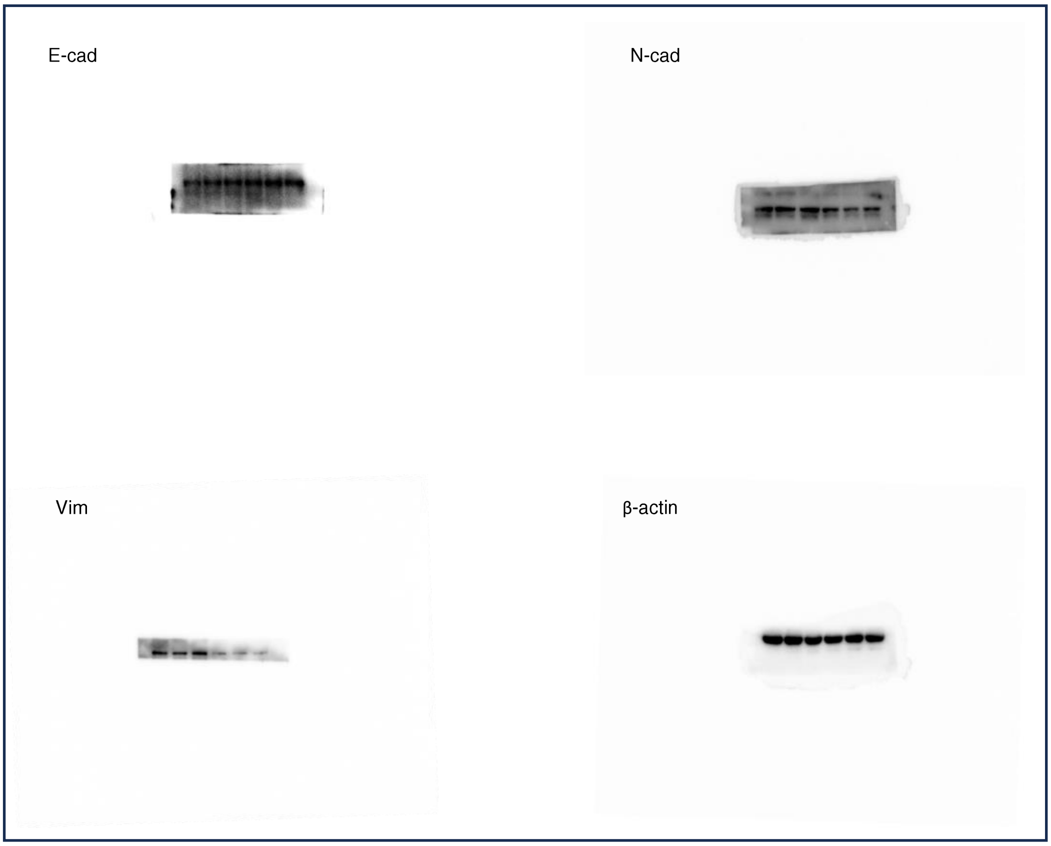

Figure 7E

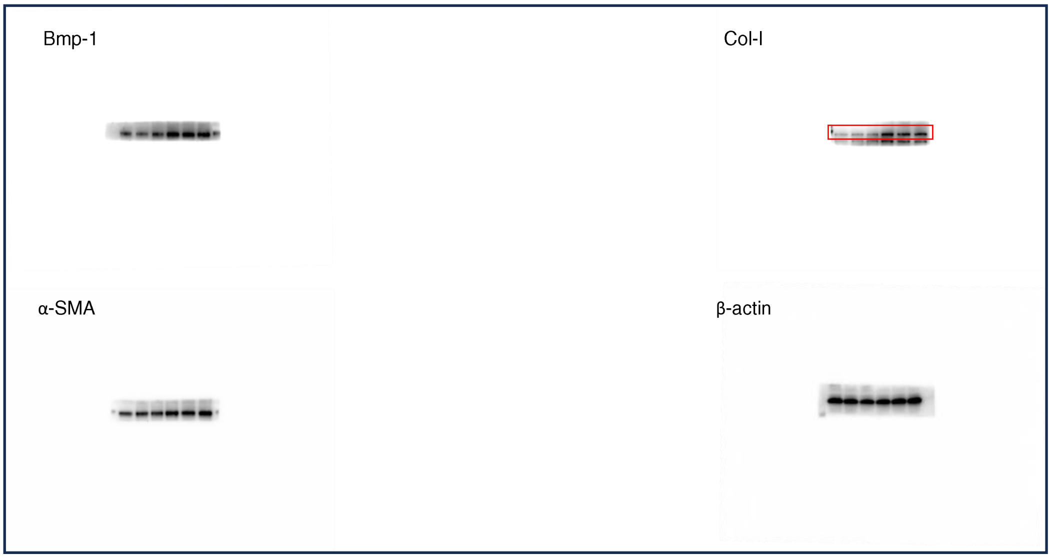

Figure 7G

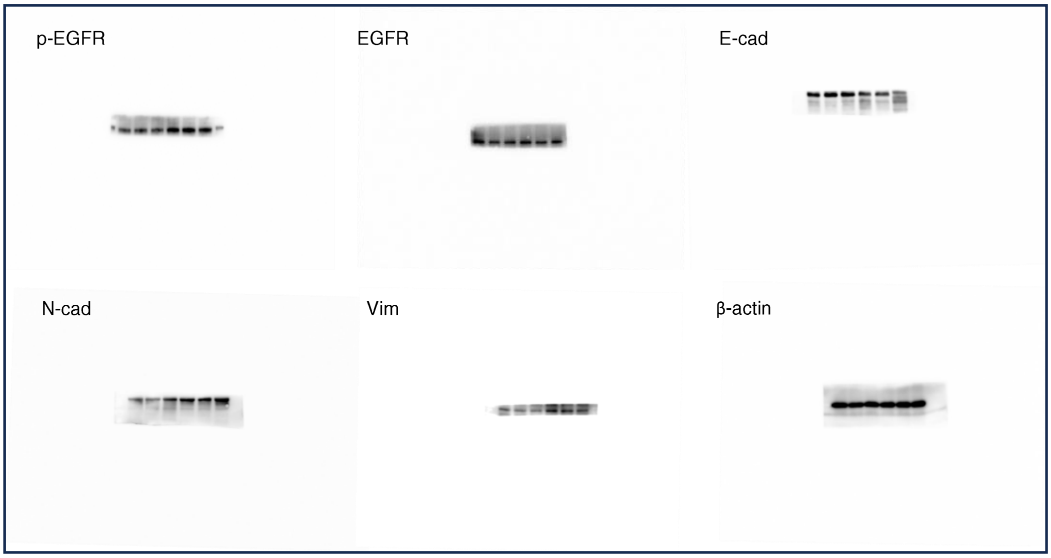

Figure 7L

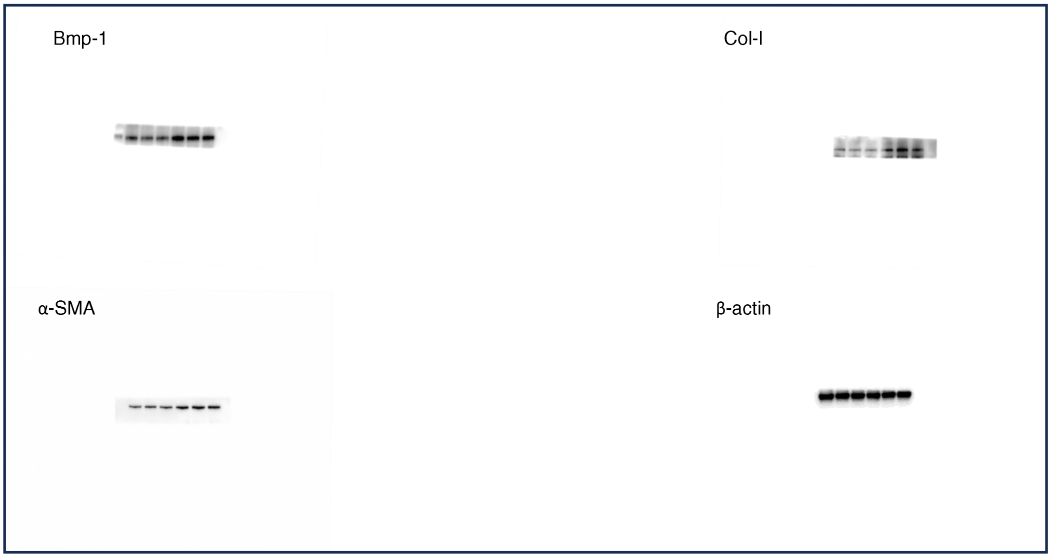

Figure 8F

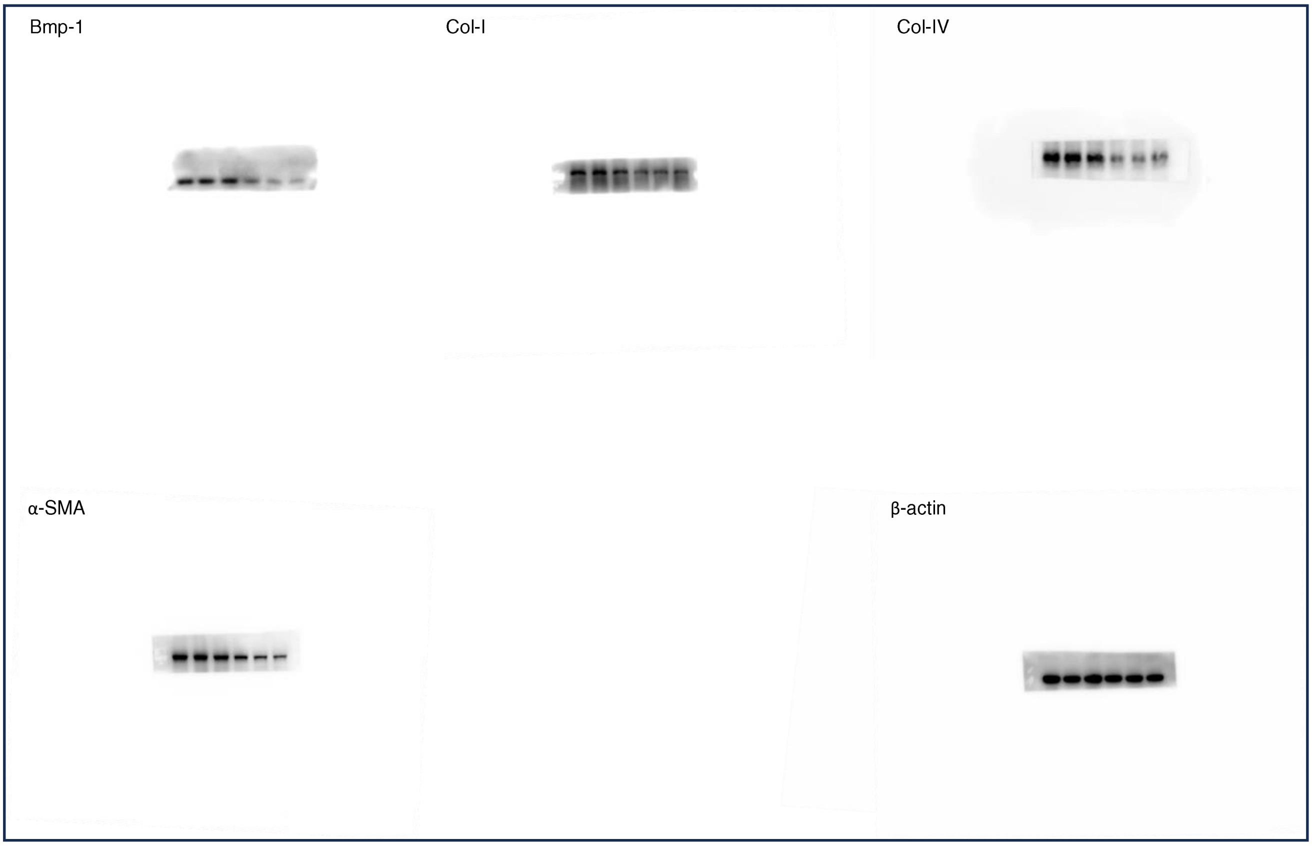

Figure 8H

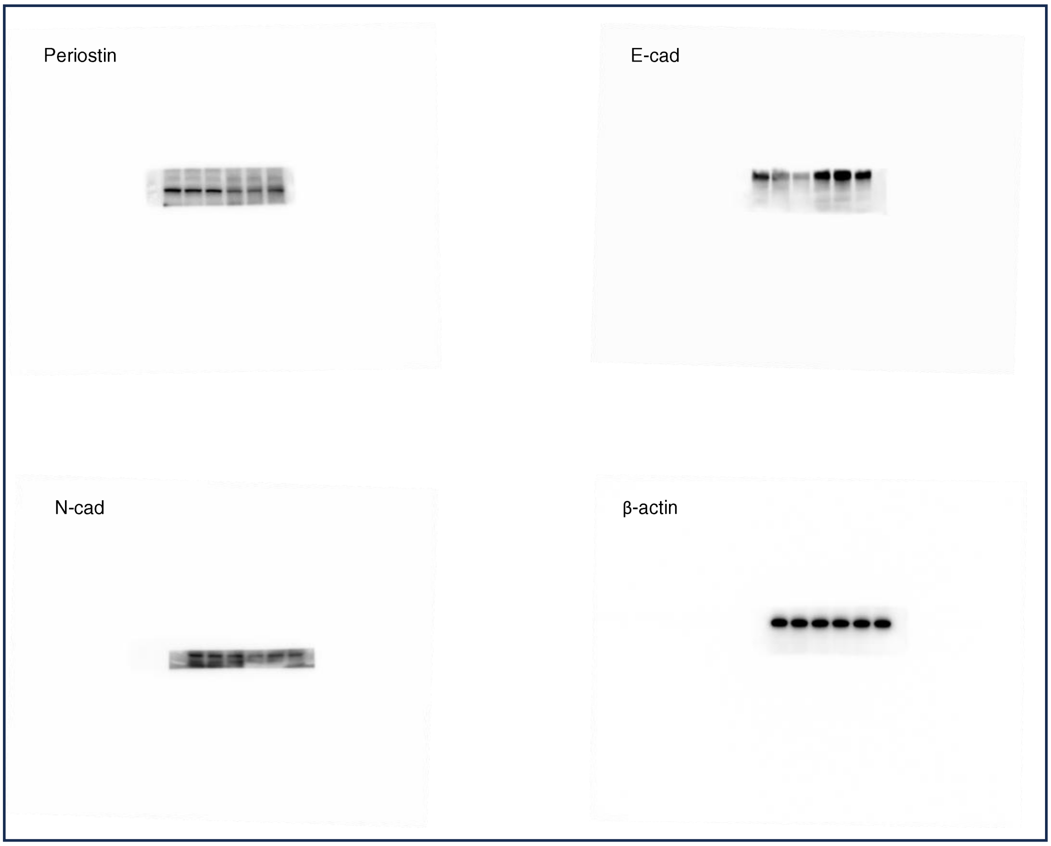

Figure 8I

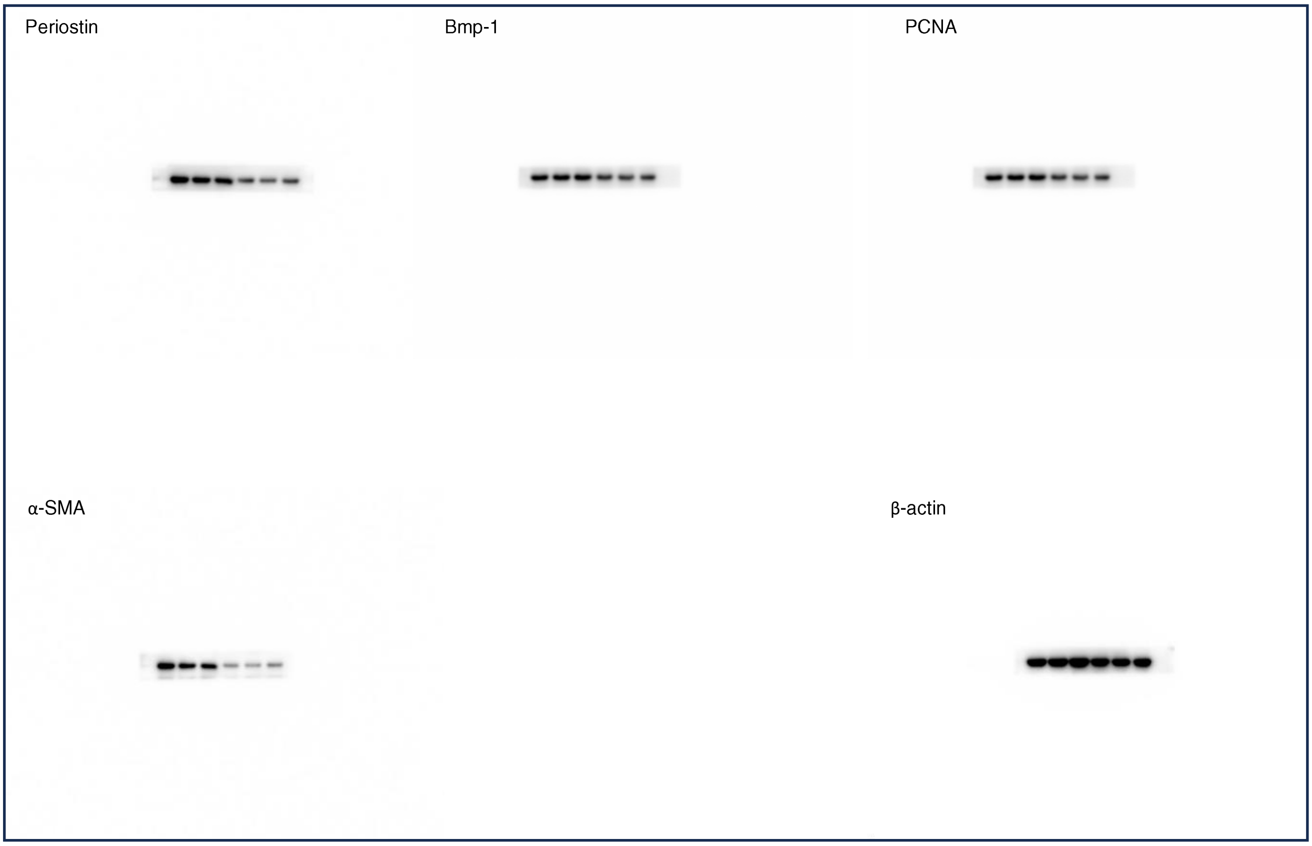

Figure 8K

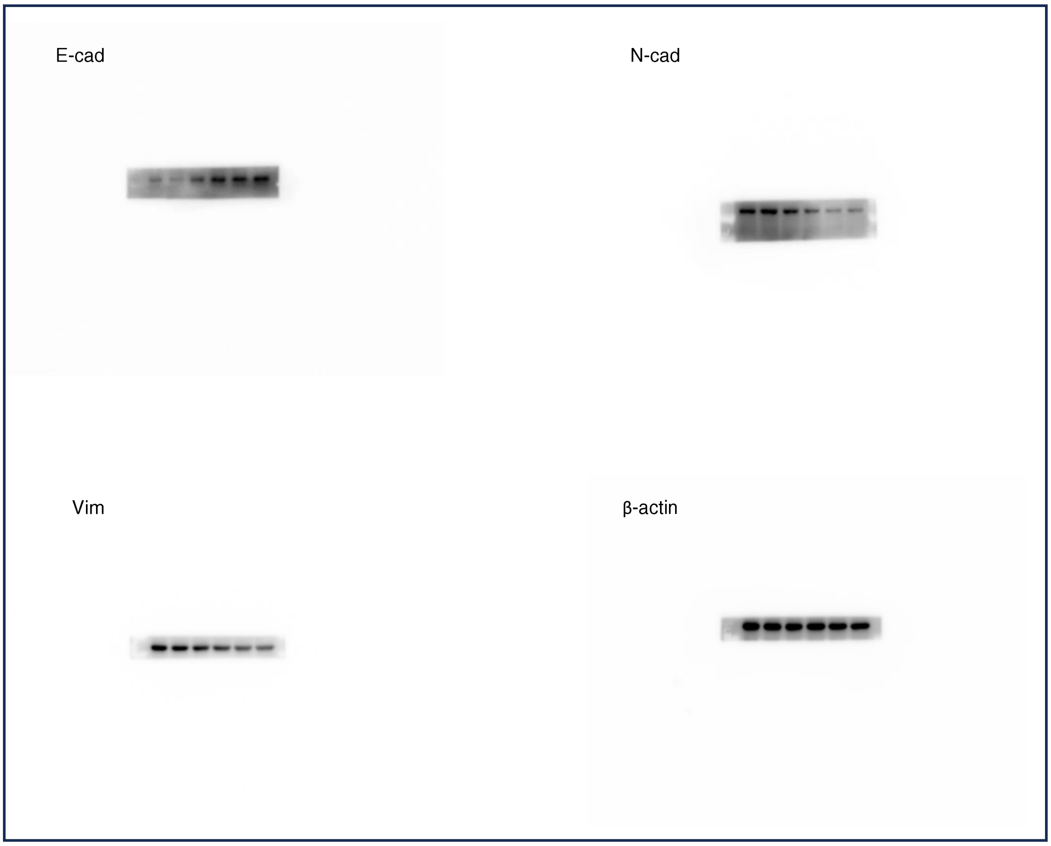

Figure S2D

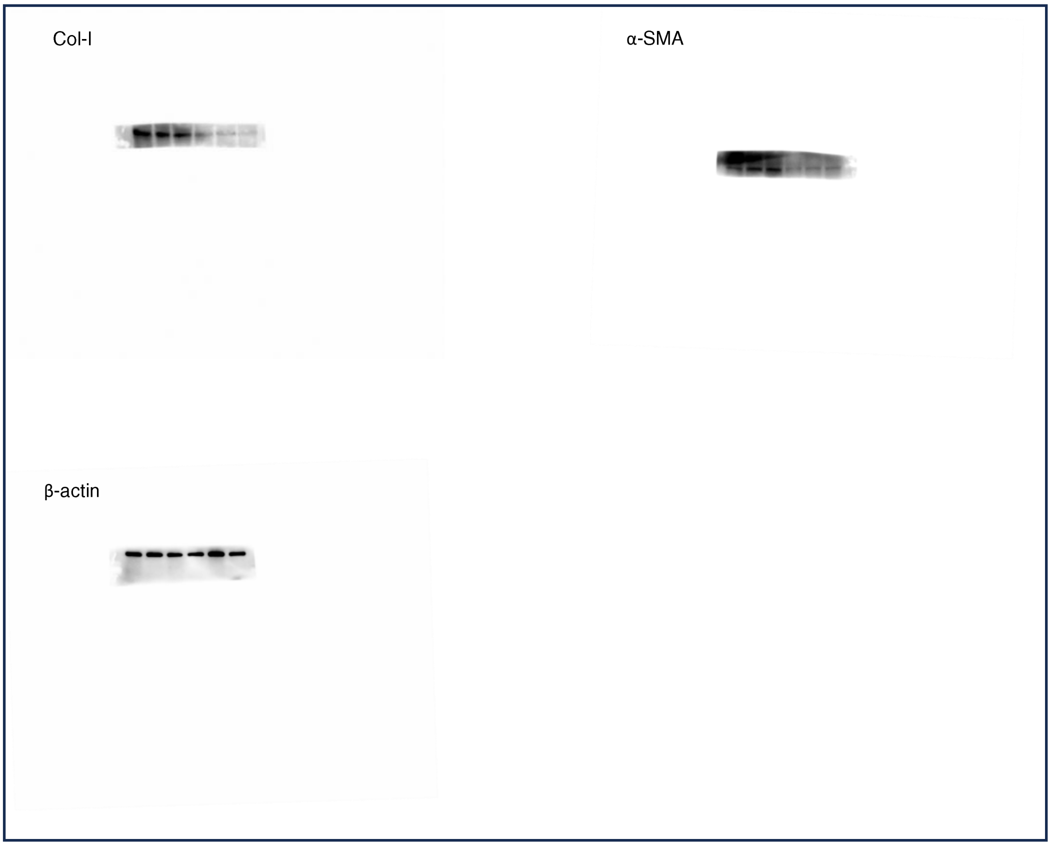

Figure S3E

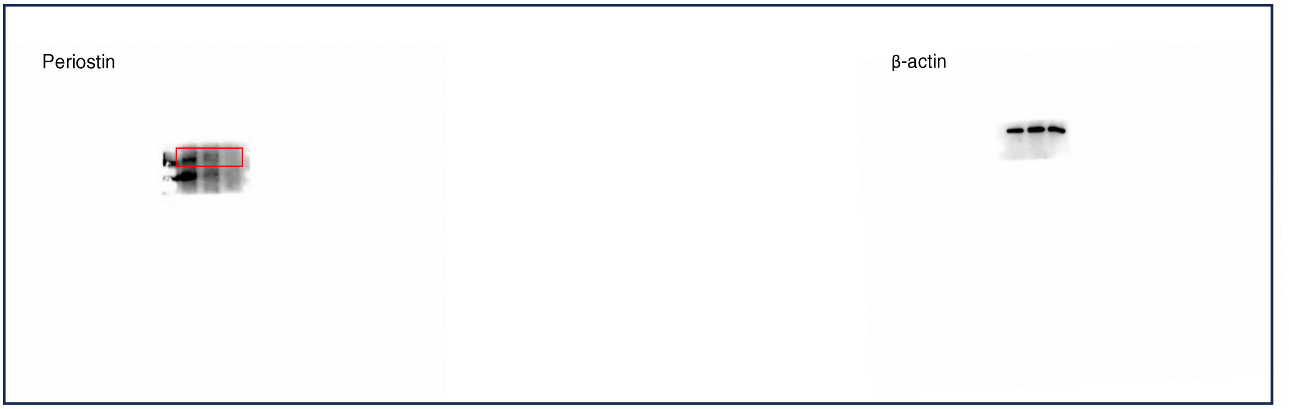

Figure S8G Left

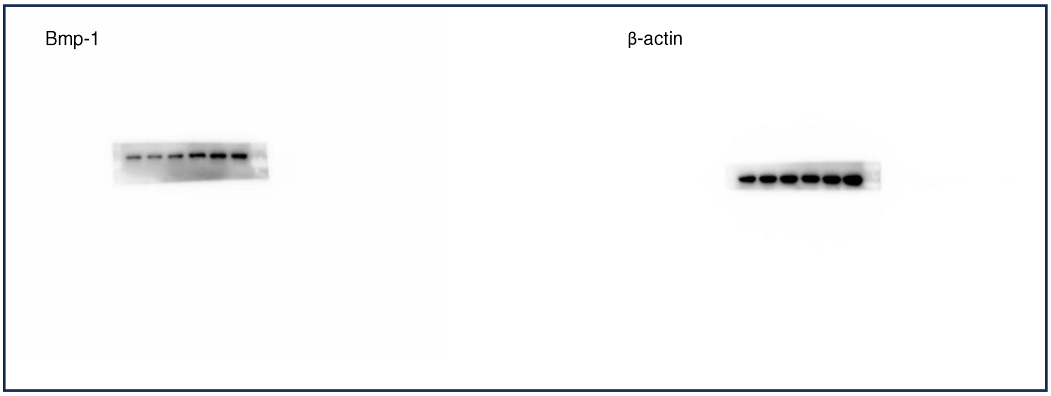

Figure S8G Right

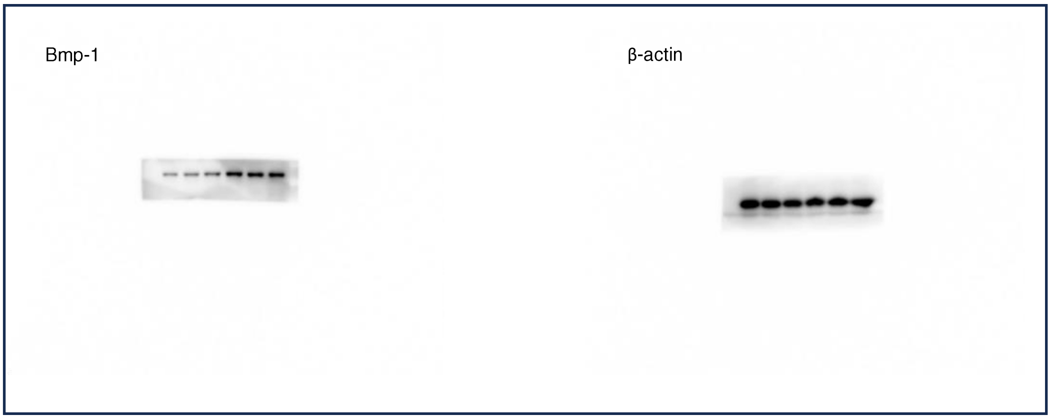

Figure S8I

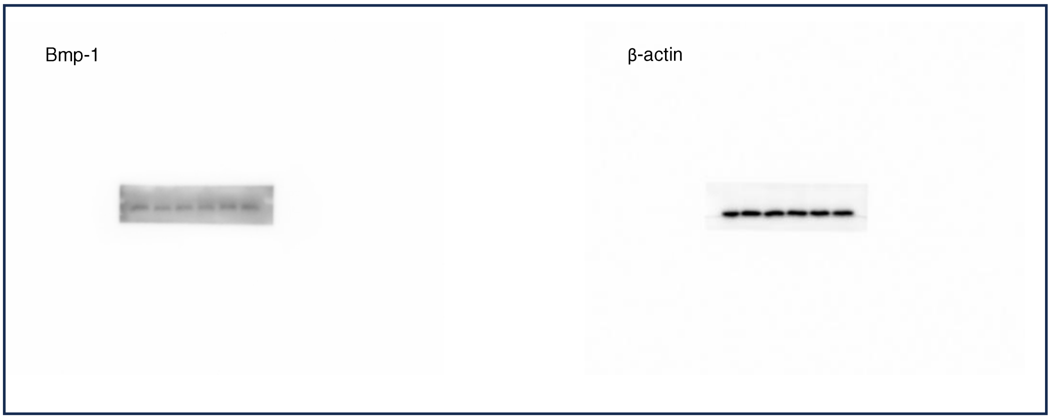

Supplement: Supplementary file 2 — Original western blots [file 41419_2024_6437_MOESM2_ESM.pdf]
